# Correlates of decisional dynamics in the dorsal anterior cingulate cortex

**DOI:** 10.1371/journal.pbio.2003091

**Published:** 2017-11-15

**Authors:** Habiba Azab, Benjamin Y. Hayden

**Affiliations:** 1 Department of Brain and Cognitive Sciences and Center for Visual Sciences, University of Rochester, Rochester, New York, United States of America; 2 Department of Neuroscience and Center for Magnetic Resonance Research, University of Minnesota, Minneapolis, Minnesota, United States of America; University of Oxford, UNITED KINGDOM

## Abstract

We hypothesized that during binary economic choice, decision makers use the first option they attend as a default to which they compare the second. To test this idea, we recorded activity of neurons in the dorsal anterior cingulate cortex (dACC) of macaques choosing between gambles presented asynchronously. We find that ensemble encoding of the value of the first offer includes both choice-dependent and choice-independent aspects, as if reflecting a partial decision. That is, its responses are neither entirely pre- nor post-decisional. In contrast, coding of the value of the second offer is entirely decision dependent (i.e., post-decisional). This result holds even when offer-value encodings are compared within the same time period. Additionally, we see no evidence for 2 pools of neurons linked to the 2 offers; instead, all comparison appears to occur within a single functionally homogenous pool of task-selective neurons. These observations suggest that economic choices reflect a context-dependent evaluation of attended options. Moreover, they raise the possibility that value representations reflect, to some extent, a tentative commitment to a choice.

## Author summary

How we make decisions based on value and how these computations are implemented in neuronal circuits remain topics of active debate. It also remains unclear how attention shapes how we perceive and process value. The dorsal anterior cingulate cortex has been implicated in both value-based decision-making and attention, although its role in these processes remains controversial. We investigated the neuronal mechanisms underlying value-based choice in the macaque dorsal anterior cingulate cortex using a task in which subjects chose between 2 sequential options. We find that value encoding in this region reflects partial commitment to a decision, suggesting this region is actively involved in the decision-making process. We also see differences in value encoding between the first- and second-viewed options, suggesting that the brain fundamentally encodes initially-viewed options differently than subsequent ones. Finally, the sequential nature of our task allowed us to falsify the hypothesis that 2 pools of neurons are linked to the 2 offers. These results point towards a comparative mechanism shaped by attention and could potentially inform future models of the neuronal circuits underlying this process.

## Introduction

When choosing between 2 options, we generally consider them in turn rather than processing them in parallel [1–6]. Sequential evaluation and comparison is necessarily favored when options are presented asynchronously but is likely to occur even when options are presented simultaneously, as we attend each one in turn. For example, when we are free to look where we like, we generally fixate on each option and preferentially evaluate the one within the fovea [1,3,7–9]. Evidence for the idea of sequential processing in choice comes from eye-movement data as well as from known limits on attention, which is normally constrained to a single focal spotlight [10–12].

Sequential choice introduces an asymmetry between the first and second option: the first option can serve as a default, which then determines the brain’s response to the second [4,13–16]. Such a context-dependent choice mechanism has been well established for memory-guided perceptual decisions [17–22]. In such decisions, the memorandum modifies the response properties of neurons so that the appearance of the probe will lead to different responses depending on their match status [23,24]. Thus, the working memory is stored in a functional, not representational, manner. We hypothesized that a similar mechanism may apply to reward-based choices.

One diagnostic feature of such a mechanism is that choice is made within a single pool of neurons. That is, the same group of cells processes both the memorandum and the probe and then also implements the comparison. In other words, the comparison involves a pool of neurons all serving the same computation. In contrast, many models of economic choice are 2-pool models [25–29]. In such models, members of each pool preferentially encode the value of a single option and compete for control of behavior. Because values are bound to corresponding options through a stable assignment of neuronal pools, we refer to these models as “labeled-line” models. At least some recent evidence suggests that such 2-pool models may not correspond to the way the brain encodes value [7,9,30].

Studies of memory-guided decisions generally use options that are uncorrelated, and thus the value of the first offer provides no information about the likely choice [17,19,21], as in some neuroeconomic studies [31]. However, in many binary choices, the first offer provides some evidence in favor or against its ultimate acceptance. Indeed, in some models of choice, decision processes for the 2 options are somewhat independent, so responses to the first offer may partially determine the ultimate decision [32]. As a consequence, if the first option is particularly good or bad, it may not matter as much what the second one is—the decision maker may take advantage of this fact and begin to compute a tentative, or partial, decision even before the second offer occurs. A signature of this process would be that the brain responds differently to the first offer, depending on whether it would later be chosen.

We recorded responses of single dorsal anterior cingulate cortex (dACC) neurons in a binary risky choice task, in which we controlled the locus of attention by presenting offers asynchronously. This region has been closely implicated in economic choice and proposed to be a key site for comparison in previous studies [33–39]. We found that population responses to the first offer were partially invariant to whether it would later be chosen, yet were also partially dependent on the upcoming decision. In contrast, responses to the second offer were wholly dependent on whether it would later be chosen. We also found that the largely overlapping populations of neurons encode each of the 2 presented offers—the opposite of what we would expect from a labeled-line architecture. This result echoes findings in other brain regions associated with value processing, including the ventromedial prefrontal cortex (vmPFC) and the ventral striatum (VS) [40,41].

## Results

### Monkeys prefer options with higher expected values

On each trial, subjects chose between 2 options presented asynchronously. Although subjects had to choose 1 of the options in order to proceed to the next trial, we will refer to each option being “accepted” or “rejected” for ease of explanation.

**Fig 1:**
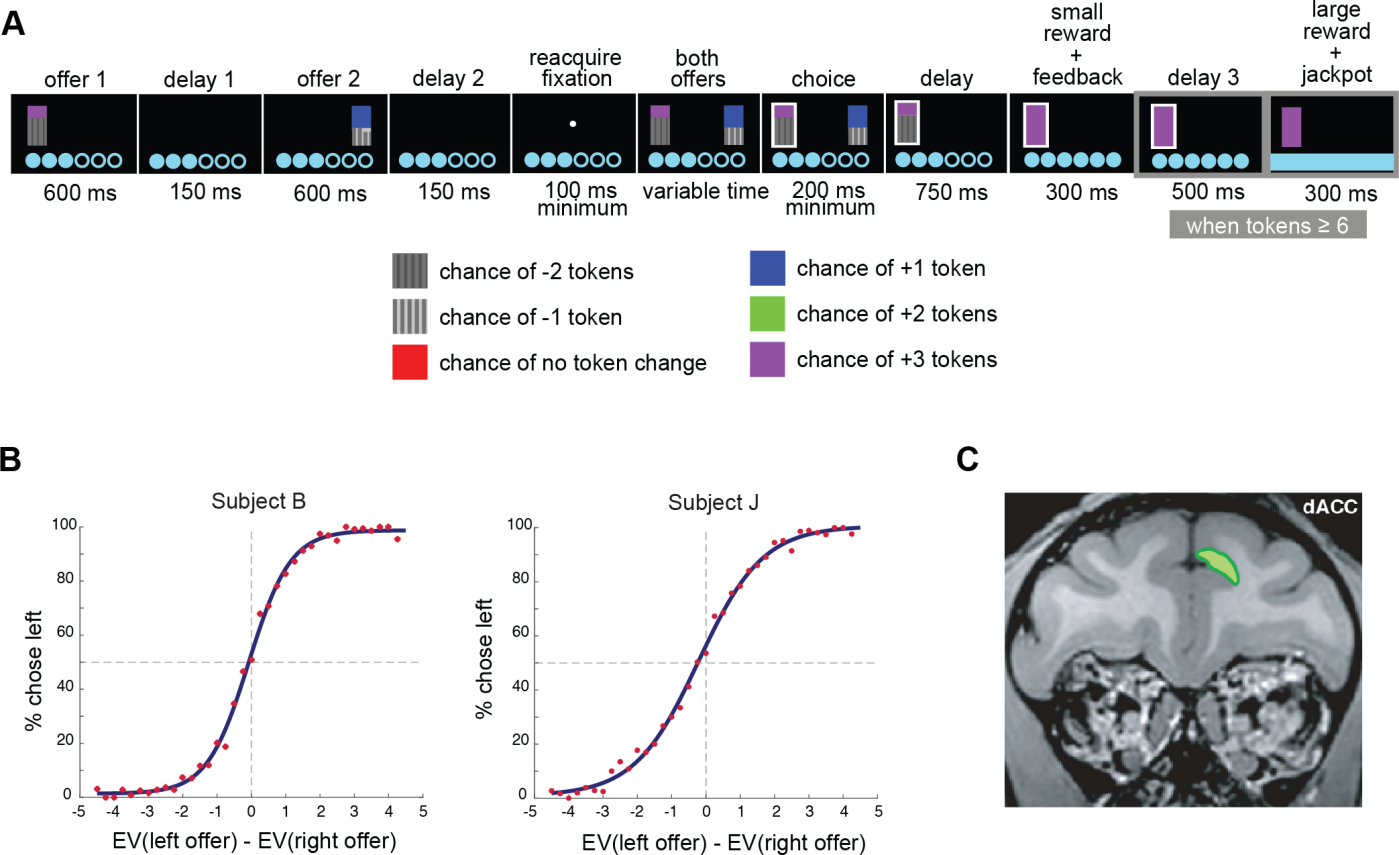
Task structure, region of interest, and summary of behavior. **(A)** Task: subjects chose between 2 asynchronously presented gambles and won or lost tokens accordingly. When subjects collected 6 or more tokens, they received a large liquid reward and token total reset to 0. **(B)** Behavior for each subject, fit to a sigmoid function. Subjects chose the left option more often as its value increased, as would be expected, given understanding of the task. **(C)** Neural responses were recorded from the dorsal banks of the anterior cingulate cortex (refer to Materials and methods). dACC, dorsal anterior cingulate cortex; EV, expected value of gamble.

Each option was a gamble defined by 3 parameters: win amount *w*, loss amount *l*, and win probability *p* (Fig 1A). These parameters were selected randomly and independently by the computer on each trial (see Materials and methods). The mathematical expected value of each offer is defined as follows:

EV=p*win+(1−p)*loss

Subjects performed this task better than chance, meaning they reliably preferred the option with greater expected value (subject B, 78.3%; subject J, 74.6%; *p* < 0.0001 on all individual sessions in both subjects, Fig 1B). Our subjects showed extremely weak spatial biases, which only reached significance for 1 subject, despite the large number of trials (subject B: 12,593 trials; subject J: 20,967 trials). Subject B chose the left option on 49.7% of trials (2-sided binomial test, *p* = 0.56), and subject J chose the left option on 51.0% of trials (2-sided binomial test, *p* = 0.0028). These biases did not appear to be influenced by trial difficulty (see S1 Text). We factor in the influence of spatial positions of the offers into future analyses by including this variable in our multiple linear regression models (see below).

To confirm that preferences depended on the values of both offers, we used a logistic regression model in which choice of the first option (versus the second) was predicted by the 2 offer values in each trial (see Materials and methods). For both subjects, aggregate regression coefficients were significantly different from 0 both for the first offer (1-sample *t* test of coefficients for offer 1 value per session: subject B, mean coefficient = 1.63, *t* stat = 16.7; subject J, mean coefficient = 1.12, *t* stat = 27.3; both *p* < 0.0001) and the second offer (subject B, mean coefficient = −1.48, *t* stat = −19.7; subject J, mean coefficient = −1.11, *t* stat = −24.0; both *p* < 0.0001). The positive and negative values for offers 1 and 2, respectively, indicate that these offers had opposite effects on the subject’s preference for the first option, as expected. These results indicate that subjects’ decisions were informed by the values of both gambles, and roughly equally so.

Subjects also made choices based on the individual parameters characterizing each gamble. Using a logistic regression model, we find that the values of the 2 possible outcomes within each gamble, as well as the probabilities of those outcomes (thus, 6 total variables), all contribute significantly towards predicting the subject’s choice (1-sample *t* test for coefficients of all 6 offer parameters: all *p* < 0.0001. See S1 Table for detailed results).

In this study, we used expected values instead of subjective values as predictors in all analyses. However, we replicated all analyses with subjective values and found that all results are qualitatively similar (see S2 Table, S3 Table, S4 Table and S5 Table).

### dACC neurons encode values of both attended and remembered offers

We recorded responses of 129 neurons in dACC (Fig 1C, also see Materials and methods) in 2 subjects. Subjects were well trained on the task (>1 month experience and consistent behavior) before recording began. In all cases, neurons were well isolated, recorded stably, and collected on single-contact electrodes.

We first quantified the encoding of each offer’s value while it was being displayed on the screen. We focused on 2 fixed epochs for this analysis, epoch 1 and epoch 2. These epochs were each 500 ms long, began 100 ms after offer 1 or 2 appeared, and ended when the offer was removed from the screen (Fig 1A). To avoid *p*-hacking or data fishing, we chose this epoch long before collecting data; specifically, it is the same one we used in previous studies using a somewhat similar task in different brain areas [40–42].

**Fig 2:**
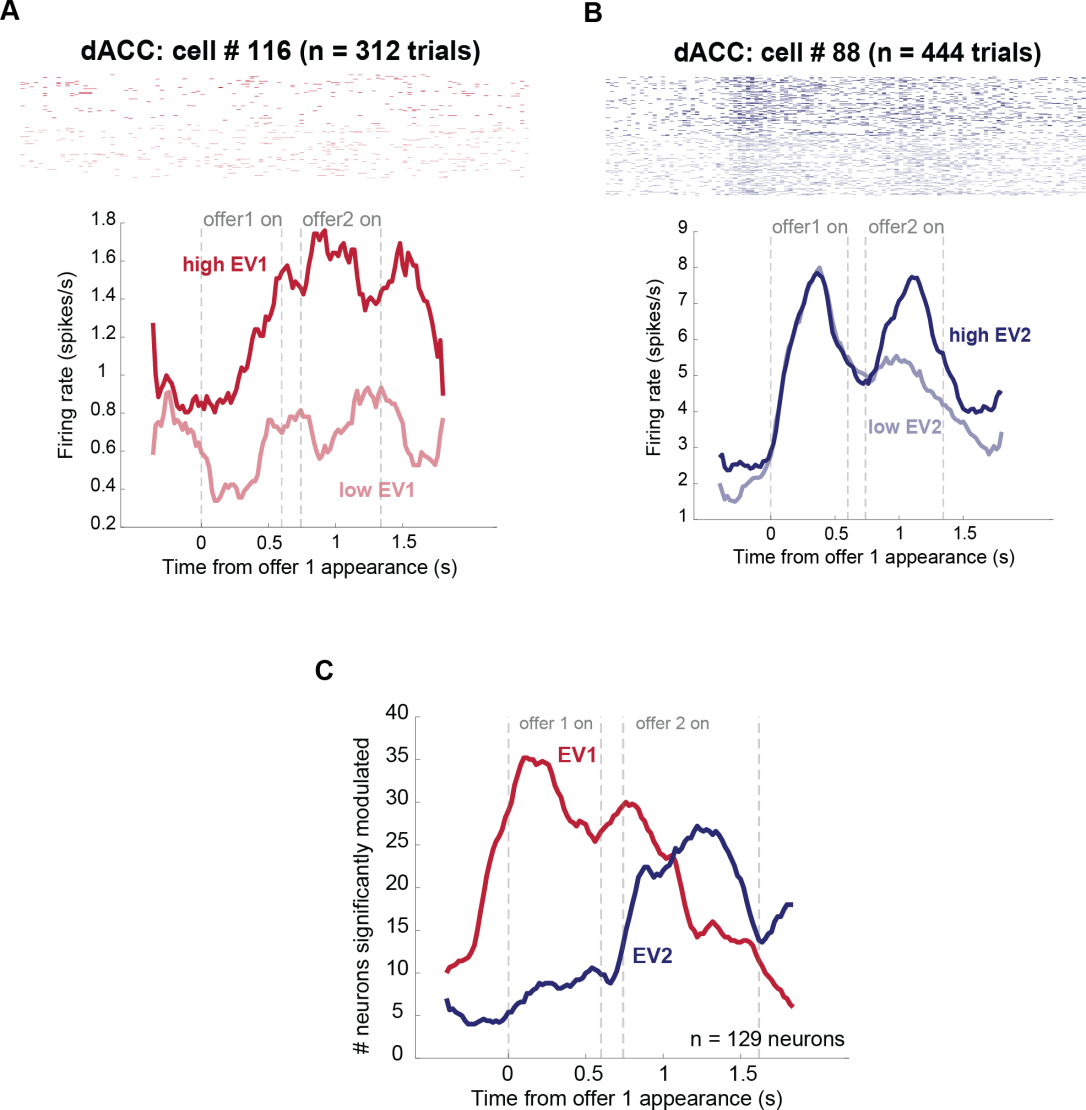
Values of the 2 offers modulate firing rates of individual dACC neurons. **(A)** Firing rate of 1 example neuron significantly modulated by the expected value of the first offer both when it was being presented (multiple linear regression: β = 0.041, *p* = 0.011) and when it was remembered (β = 0.051, *p* = 0.0097). **(B)** Example dACC neuron activity, significantly modulated by the value of the second offer while it was being presented (β = 0.046, *p* = 0.0061). **(C)** Percentage of neurons significantly tuned to the value of the first (red) and second (blue) offers through the course of the trial, using a multiple linear regression with a sliding 500-ms window. *Data used to generate these plots can be found at https://doi.org/10.5061/dryad.h52f8*. dACC, dorsal anterior cingulate cortex; EV, expected value of gamble.

We used a multiple linear regression model in which firing rate is predicted by offer values, as well as 3 other variables: (1) the number of tokens currently collected, (2) the side the first offer appears on, and (3) the chosen offer side. We included these 3 additional parameters to control for any variability in firing rates they account for but do not further discuss them in this study. Fig 2A and 2B show 2 example cells, the firing rates of which were affected by offer values in the first and second epochs.

In our sample of cells, 27.1% (35/129) of neurons encoded the expected value of the first offer during the first epoch (that is, while it appeared on the screen: 2-sided binomial test, *p* < 0.0001). During the second epoch, a similar but slightly lower proportion, 23.3% (30/129), of neurons encoded the same offer 1 value (*p* < 0.0001). This finding suggests that the offer 1 value was retained, presumably in working memory, even when it was no longer on the screen. During the second epoch, 16.3% (21/129) of neurons encoded the value of the second offer (*p* < 0.0001). We see no significant bias towards positive/negative encoding of any of these values, neither in the population of significantly tuned cells (*p* > 0.8 in all cases) nor in the overall population (*p* > 0.4 in all cases). Note that these positive/negative bias statistics are not corrected for multiple comparisons; had we done so, effects would be even further from significance. All analyses yielded the same qualitative results when subjective values for offers were used instead of expected values (see S2 Table and S3 Table).

We also examined responses to the individual offer components; namely, the higher and lower outcomes and the probability of the higher outcome (the probability of the lower outcome is deterministically 100% minus the probability of the higher outcome). We looked at responses to offer 1 during the first epoch, when nothing else has been presented during this trial. The proportion of neurons selective for the probability of the offer 1 higher outcome was 26/129 (20.2%, *p* = 1.1756 × 10^−9^); the proportion selective for the value of the large payoff for offer 1 was 21/129 (16.3%, *p* = 1.8453 × 10^−6^), and the proportion selective for the value of the lower outcome was 12/129 (9.30%, *p* = 0.0300).

Finally, we looked at the variance explained by each of the offers’ values as a fraction of the variance explained by task variables as a whole. Offer 1 value explains, on average, 17.2% of total variance explained by task-relevant variables across the entire population in epoch 1 and 14.8% in epoch 2. Offer 2 explains an average of 13.9% of total variance explained across the population in epoch 2.

### Offer value encoding is decision dependent throughout the trial

#### Encoding of first offer while attended includes both choice-dependent and choice-independent elements

Here, we use the term “format” as shorthand to refer to a population’s ensemble tuning function for a particular variable [43]. By our definition, it consists of a vector of linear regression coefficients, 1 coefficient for each neuron, indicating the linear component of that neuron’s modulation in response to the variable in question (see Materials and methods for further details). This measure resembles other aggregate tuning measures used in previous studies [44–48]. Our approach here improves on one we used previously by (1) using multiple linear regression, which controls for other variables that may affect firing rates, and (2) taking into account noise in our estimate of each neuron’s modulation by different task variables.

Specifically, we use a Bayesian regression model to obtain a distribution of potential regression coefficients for every neuron, rather than a single value. This procedure produces a matrix of coefficients with multiple estimates for each neuron in the population. This in turn allows us to estimate the underlying distribution of the correlation between the encoding of different variables, while taking into account noise at the individual neuron level, in order to visualize the spread of the distribution of this statistic. We compared this distribution to one generated at random by shuffling trials randomly, regardless of which option was ultimately chosen (i.e., a permutation test). If ensemble responses truly carry no information about the upcoming decision (and differ across trials only due to noise), this distribution generated from randomly shuffled trials should overlap significantly with that generated by splitting trials according to which offer was accepted. In contrast, if the upcoming decision does affect value encoding, coding formats for to-be-accepted and to-be-rejected offers should be less correlated than expected only due to noise; the true correlation coefficient should be significantly smaller than that generated through a random shuffling of trials.

**Fig 3:**
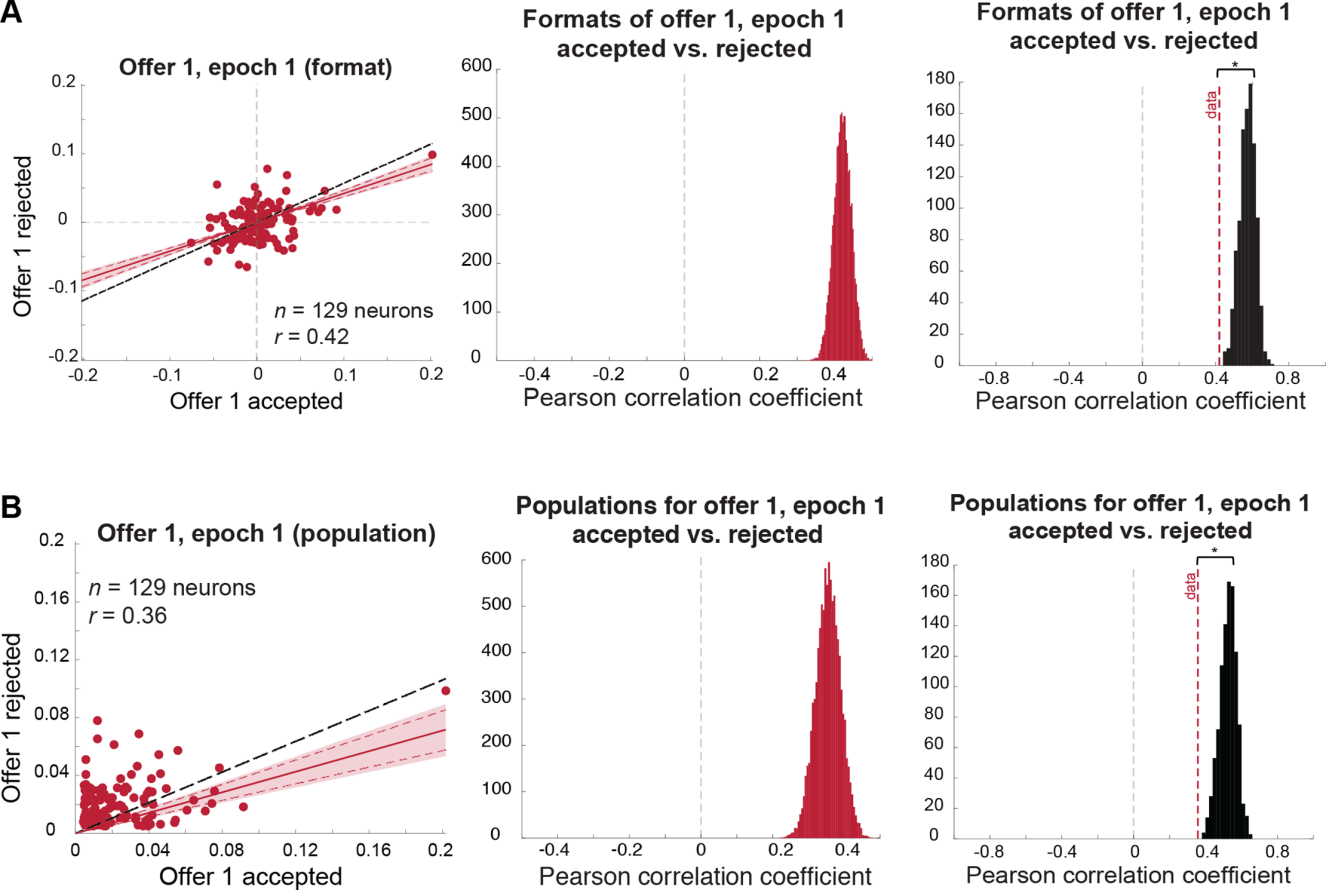
Coding format for offer 1 in the first epoch is similar, regardless of whether it will be accepted or rejected, yet also weakly depends on the upcoming decision. Results also hold when the outlier cell is removed. **(A)** Format analysis: (left) scatterplot of offer 1/epoch 1 regression coefficients for trials in which offer 1 is accepted (horizontal axis) versus rejected (vertical axis). Shaded error region indicates 99% credible interval, and dashed red lines indicate the 95% credible interval. The black dashed line indicates the level of correlation we would expect to see under a chance model (see main text and Materials and methods). (Middle) distribution of mean correlation coefficient of data. The mean of this distribution is represented as a red dashed line in the right panel. (Right) distribution of mean correlation coefficients expected under a chance model (black distribution) compared to the mean correlation observed in the data (red dashed line; permutation test with 1,000 permutations: *p* < 0.001). **(B)** Population analysis: (left) scatterplot of offer 1/epoch 1 absolute regression coefficients for trials in which offer 1 is accepted (horizontal axis) versus rejected (vertical axis). Shaded error region indicates 99% credible interval, and dashed red lines indicate the 95% credible interval. The black dashed line indicates the level of correlation we would expect to see under a chance model (see main text and Materials and methods). (Middle) distribution of mean correlation coefficient of data. The mean of this distribution is represented as a red dashed line in the right panel. (Right) distribution of the mean correlation coefficients expected under a chance model (black distribution) compared to the mean correlation observed in the data (red dashed line; permutation test with 1,000 permutations: *p* < 0.001). *Data used to generate these plots can be found at https://doi.org/10.5061/dryad.h52f8*.

We compared coding formats for the values of accepted and rejected first offers during the presentation of the first offer (epoch 1). These formats are positively correlated (mean Pearson correlation: *r* = 0.42, 99% credible interval: [0.36, 0.48]; Fig 3A, left and middle panels). The positive value indicates that the way in which these neurons signal value is, to some degree, independent of whether the option will later be chosen.

Nonetheless, this correlation, while positive, is not as strong as would be expected if there were no difference between accept and reject trials except for that due to noise. We can estimate this ceiling measure (which would ideally be *r* = 1 but in our data is *r* = 0.57 due to trial-by-trial noise; Fig 3A, right panel, black distribution) by shuffling the labels on the trials at random. The correlation we observe in our data is significantly smaller than this ceiling value (permutation test: *p* < 0.001; Fig 3A, right panel). These results indicate a simultaneous dependence on and independence of the upcoming decision, consistent with the idea that the ensemble of neurons reflects a partially completed decision.

Note that the difference in coding schemes for accepted and rejected offers is unrelated to the fact that accepted offers have higher values and rejected ones have lower values. Our analyses compare indices of modulation (the regression coefficients) rather than raw firing rates. If a neuron’s firing rate carries no information about the upcoming decision, this regression coefficient should be the same, independent of offer value, and estimating it from different trials should yield highly correlated values (as shown by the same analysis on shuffled trials; Fig 3A, right panel, black distribution). Therefore, our results are not due to the correlation between offer value and likelihood of accepting an offer.

We next sought to determine whether the value of the first offer, when it was later accepted versus rejected, was encoded in overlapping or discrete subpopulations of neurons in our sample. We did this using the same general approach detailed above (for more detail, see Materials and methods), except using the absolute (rather than signed) regression coefficients [43]. This allowed us to compare the strength of modulation for each neuron across conditions while ignoring direction. Using this analysis, we found that the populations of cells that encode a to-be-chosen offer 1 and a to-be-rejected offer 1 overlap more than expected by chance (Pearson correlation of absolute regression coefficients: *r* = 0.36, 99% credible interval: [0.27, 0.44]). Specifically, these subpopulations overlap more than expected for random neuronal assignment (i.e., *r* is approximately 0) or for distinct populations (i.e., *r* < 0) [43]. Nonetheless, the populations were also demonstrably not perfectly overlapping. Specifically, this correlation is significantly weaker than we would expect with perfect overlap (i.e., ceiling measure *r* = 0.53; permutation test: *p* < 0.001). Thus, the neurons most strongly involved in encoding offer 1 value change a bit depending on whether it will later be chosen.

#### Encoding of first offer in the second epoch is more dependent on the upcoming decision

**Fig 4:**
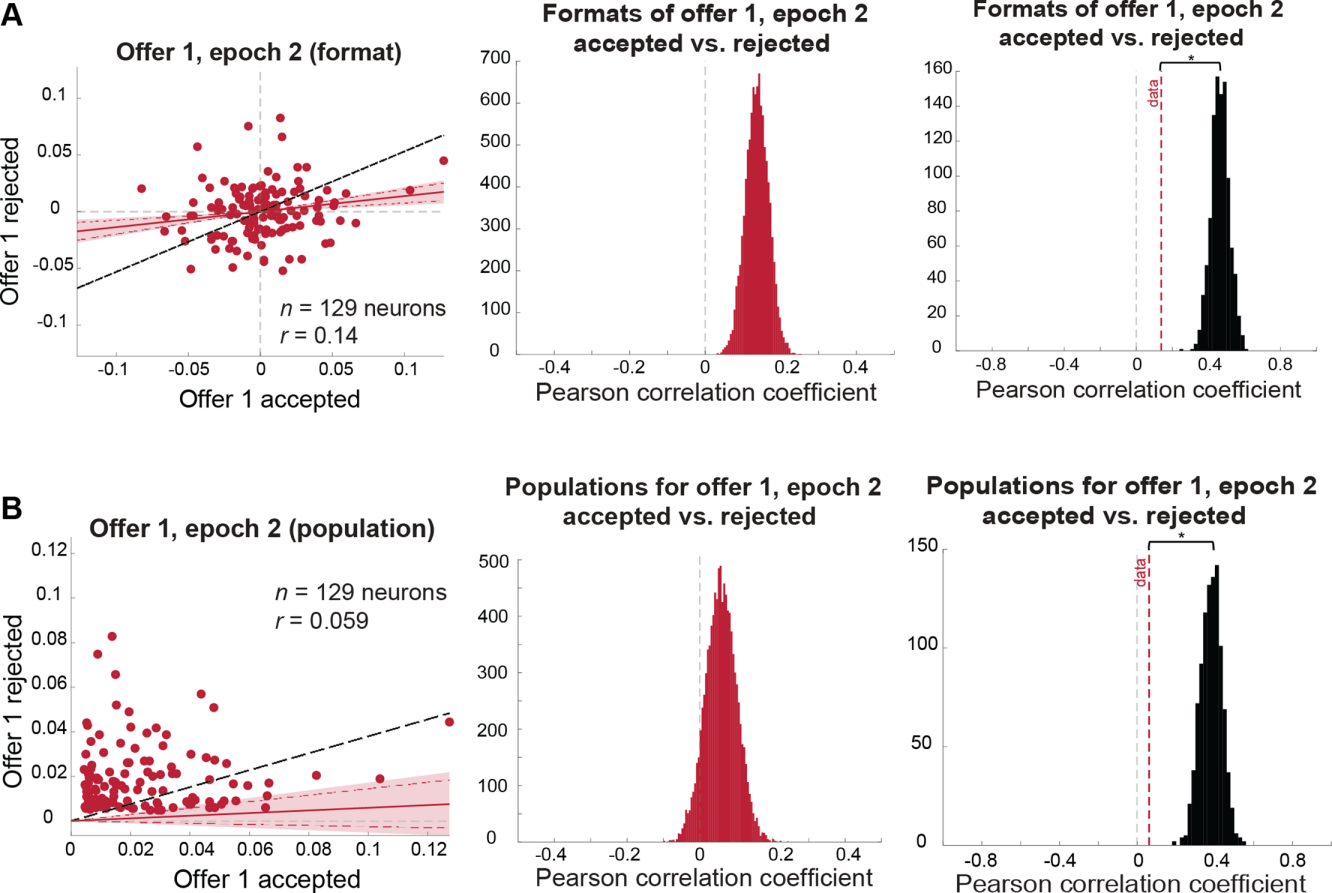
Coding of offer 1 in the comparison epoch remains correlated across accept/reject conditions but with more dependence on the upcoming decision (for statistical comparison between format correlations, see Fig 5). **(A)** Format analysis: (left) scatterplot of offer 1, epoch 2 regression coefficients for trials in which offer 1 is accepted (horizontal axis) versus rejected (vertical axis). Shaded error region indicates 99% credible interval, and dashed red lines indicate the 95% credible interval. The black dashed line indicates the level of correlation we would expect to see under a chance model (see main text and Materials and methods). (Middle) distribution of mean correlation coefficient of data. The mean of this distribution is represented as a red dashed line in the right panel. (Right) distribution of mean correlation coefficients expected under a chance model (black distribution) compared to the mean correlation observed in the data (red dashed line; permutation test with 1,000 permutations: *p* < 0.001). **(B)** Population analysis: (left) scatterplot of offer 1, epoch 2 absolute regression coefficients for trials in which offer 1 is accepted (horizontal axis) versus rejected (vertical axis). Shaded error region indicates 99% credible interval, and dashed red lines indicate the 95% credible interval. The black dashed line indicates the level of correlation we would expect to see under a chance model (see main text and Materials and methods). (Middle) distribution of mean correlation coefficient of data. The mean of this distribution is represented as a red dashed line in the right panel. (Right) distribution of mean correlation coefficients expected under a chance model (black distribution) compared to the mean correlation observed in the data (red dashed line; permutation test with 1,000 permutations: *p* < 0.001). *Data used to generate these plots can be found at https://doi.org/10.5061/dryad.h52f8*.

**Fig 5:**
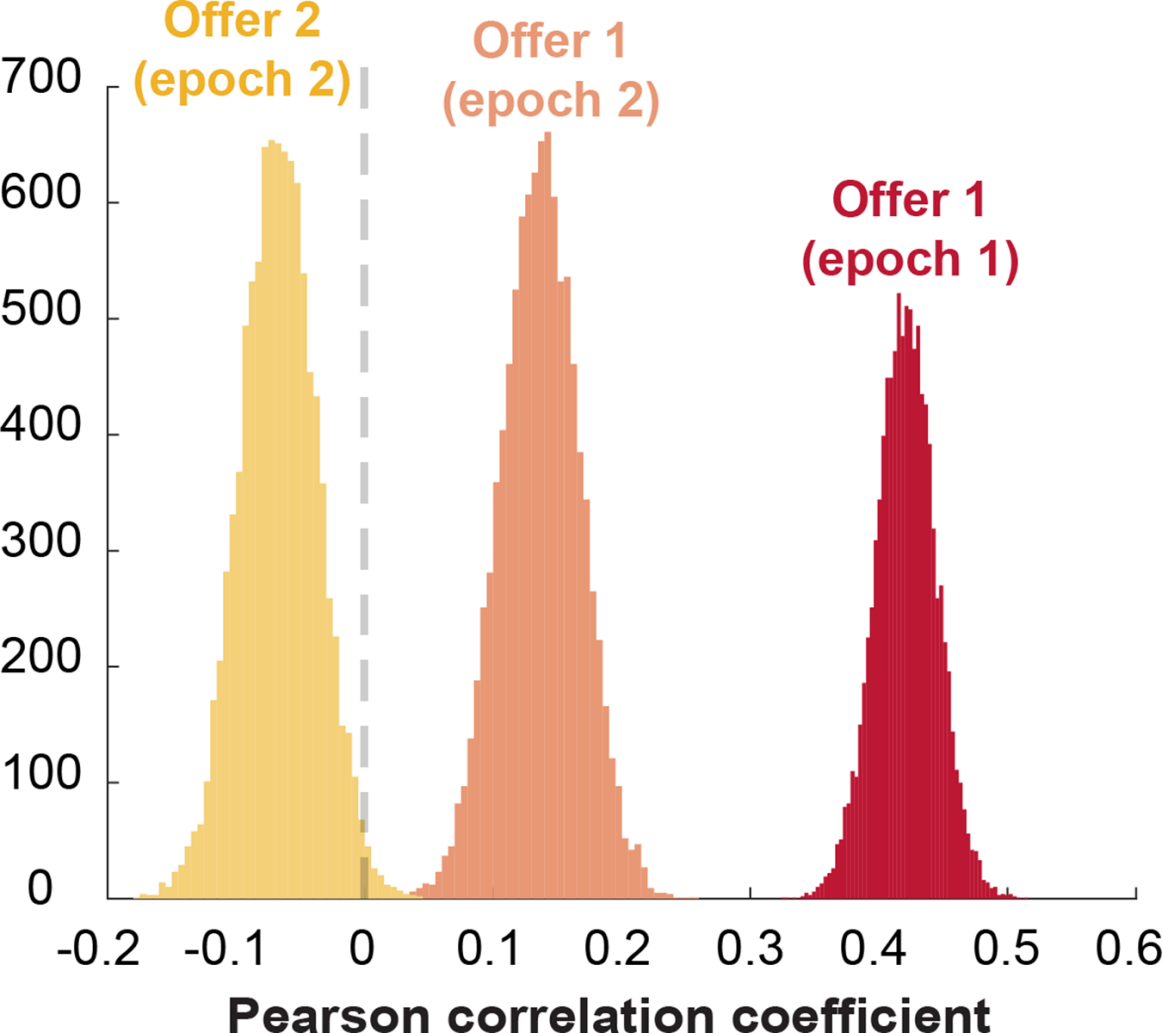
Encoding formats of offer value when the offer is later accepted versus rejected become successively more separable. Shown above are the distributions of correlation coefficients between encoding formats of offers when they were later accepted versus rejected, for offer 1 in the first and second epochs and offer 2 in the second epoch. These distributions are all significantly different from each other (statistics reported in main text). *Data used to generate these plots can be found at https://doi.org/10.5061/dryad.h52f8*.

We next performed the above analyses on offer 1 responses in epoch 2, when offer 2 was being presented, and offer 1 was stored in working memory (epoch 2). By this point in the trial, the subject possessed all the information necessary to make a choice. We therefore expected the encoding of the value of the first offer to be more dependent on the upcoming decision. Indeed, coding formats were still positively correlated (Pearson correlation: *r* = 0.14, 99% credible interval: [0.056, 0.22]; Fig 4A, left and middle panels) and still less correlated than would be expected for a signal that is entirely independent of the decision (i.e., ceiling measure *r* = 0.47; permutation test: *p* < 0.001; Fig 4A, right panel). This measure of similarity between formats was also significantly smaller than in the first epoch, indicating greater dependence on the upcoming decision (Kolmogorov-Smirnov [KS] test on distributions of correlation coefficients from epochs 1 and 2: KS stat = 1.0, *p* < 0.0001; Fig 5). In other words, coding formats measurably diverged when moving from the first to the second epoch, as if the partial decision were further along the process of completion during the second epoch.

Unlike in the first epoch, the future choice markedly changed the populations encoding the value of the first offer in epoch 2. Indeed, those populations became statistically indistinguishable from orthogonal (Pearson correlation of absolute regression coefficients: *r* = 0.059, 99% credible interval: [−0.051, 0.17]). This lack of a significant correlation is likely not solely due to a lack of signal in our data, because it is significantly weaker than the correlation we can detect under a chance model (in which trials are shuffled across conditions: ceiling measure *r* = 0.38; permutation test: *p* < 0.001). However, this is not to say these populations are separate, in which case, we would observe a significant negative correlation (i.e., neurons encoding accepted offer values are less likely to encode rejected offer values). This result is consistent with the idea that moving from the first to the second offer makes ensemble coding more distinct, again, likely because the decision is further along and thus more developed.

#### Ensemble coding of second offer depends wholly on choice

**Fig 6:**
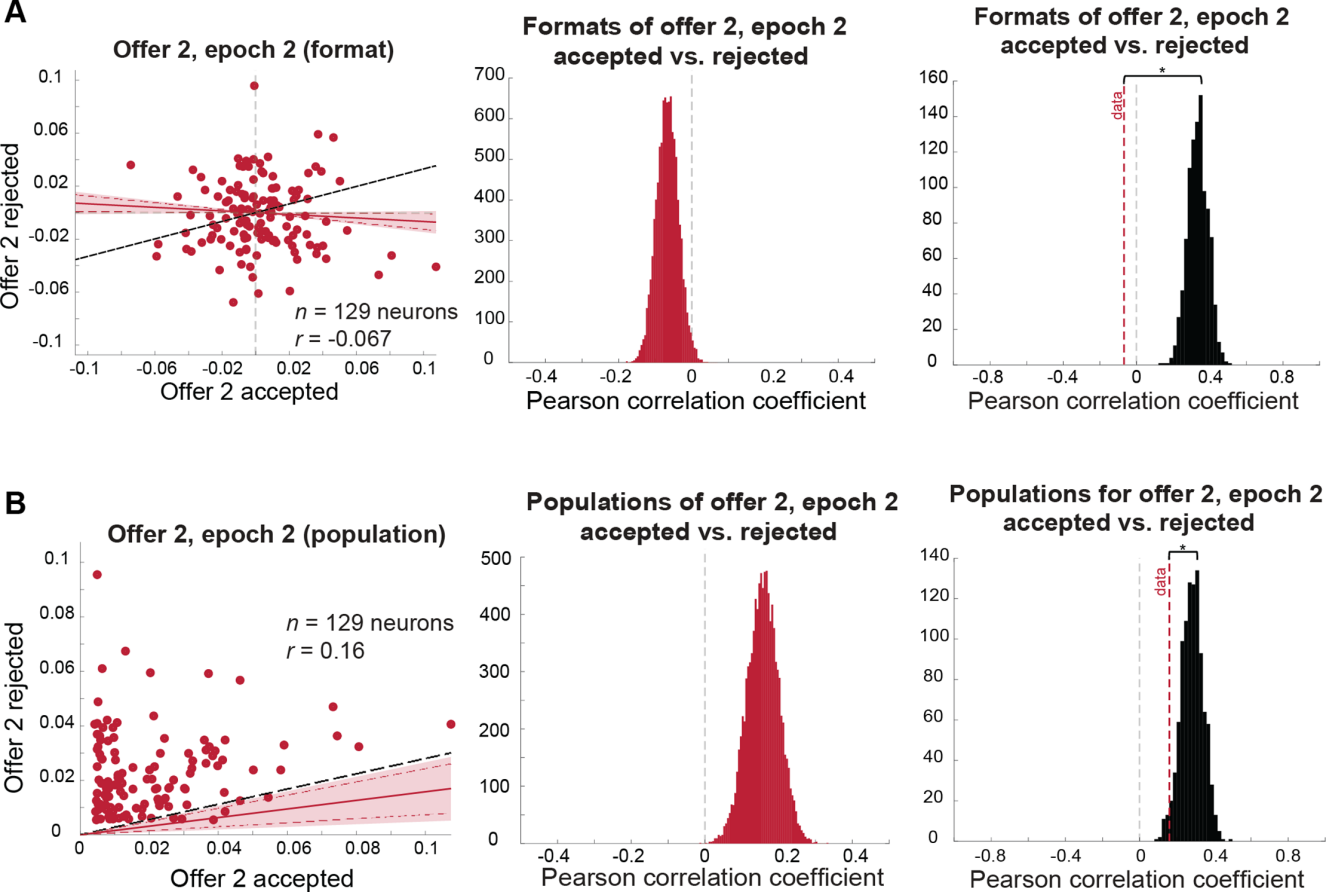
Coding of offer 2 in the comparison epoch is dependent on the upcoming decision (for statistical comparison between format correlations, see Fig 5). Red lines indicate mean correlation, red shaded area indicates 99% credible intervals, red dashed lines indicate 95% credible intervals, and black dashed line indicates the correlation expected under a chance model. **(A)** Format analysis: (left) scatterplot of offer 2, epoch 2 regression coefficients for trials in which offer 2 is accepted (horizontal axis) versus rejected (vertical axis). (Middle) distribution of mean correlation coefficient of data. The mean of this distribution is represented as a red dashed line in the right panel. (Right) distribution of mean correlation coefficients expected under a chance model (black distribution) compared to the mean correlation observed in the data (red dashed line; permutation test with 1,000 permutations: *p* < 0.001). **(B)** Population analysis: (left) scatterplot of offer 2, epoch 2 absolute regression coefficients for trials in which offer 2 is accepted (horizontal axis) versus rejected (vertical axis). Shaded error region indicates 99% credible interval and dashed red lines indicate the 95% credible interval. The black dashed line indicates the level of correlation we would expect to see under a chance model (see main text and Materials and methods). (Middle) distribution of mean correlation coefficient of data. The mean of this distribution is represented as a red dashed line in the right panel. (Right) distribution of mean correlation coefficients expected under a chance model (black distribution) compared to the mean correlation observed in the data (red dashed line; permutation test with 1,000 permutations: *p* = 0.026). *Data used to generate these plots can be found at https://doi.org/10.5061/dryad.h52f8*.

We then performed the same analyses on the encoding of the value of the second offer while it was presented (epoch 2). We found that dACC neurons use orthogonal formats on trials when it is accepted and when it is rejected (Pearson correlation: *r* = −0.067, 99% credible interval: [−0.15, 0.011]; Fig 6A, left and middle panels). This anticorrelation is not significantly different from zero and is significantly less than the value that would be expected if dACC were blind to choice (i.e., ceiling measure *r* = 0.33; permutation test: *p* < 0.001; Fig 6A, right panel). This measure provides some assurance that the lack of correlation is not simply due to the fact that our data are too noisy to detect existing correlations. Not surprisingly, the near-zero correlation we do observe is significantly weaker than the analogous correlations observed for offer 1 in both the first and second epochs (KS test: offer 1, epoch 1: KS stat = 1.0, *p* < 0.0001; offer 1, epoch 2: KS stat = 0.9992, *p* < 0.0001; Fig 5).

We next examined whether the offer 2 value is encoded in the same or different populations according to whether it is later chosen or not. While the populations of neurons encoding the offer 2 value are somewhat overlapping (Pearson correlation of absolute regression coefficients: *r* = 0.16, 99% credible interval: [0.049, 0.27]), this correlation is significantly smaller than we would expect if the populations were strictly identical (ceiling measure *r* = 0.28; permutation test: *p* = 0.026). This result indicates that the subset of neurons encoding the value of offer 2 on accept and reject trials overlaps more than would be expected by chance.

### Value comparison and attention shape responses of dACC neurons

In the following section, we test 3 hypotheses predicted by a 2-pool neural architecture underlying value comparison in the context of a sequential choice task (based on Wang’s 2002 2-pool model, with recurrent excitation and mutual inhibition between pools [49]). First, we test for putative correlates of mutual inhibition between offer values during comparison. This feature has been observed in other regions associated with value processing (including the vmPFC [40,50] and the VS [41]) but is not sufficient to confirm an underlying 2-pool architecture. The remaining 2 hypotheses can only be tested in the context of a sequential task (or at least one where options are attended sequentially and the experimenter can determine which option is attended at each point in time). Second, we test whether the first offer’s value is retained in the same population of neurons and encoded in the same format, as would be predicted by 2-pool models. Third, we test for mutual inhibition through time. This hypothesis predicts that the first offer (when attended) and the second offer (when attended) will be encoded in opposing formats. This implies that neurons encode these offers’ values in opposing formats throughout the trial, not only during comparison.

#### Testing hypothesis 1: Mutual inhibition between offer values during comparison

**Fig 7:**
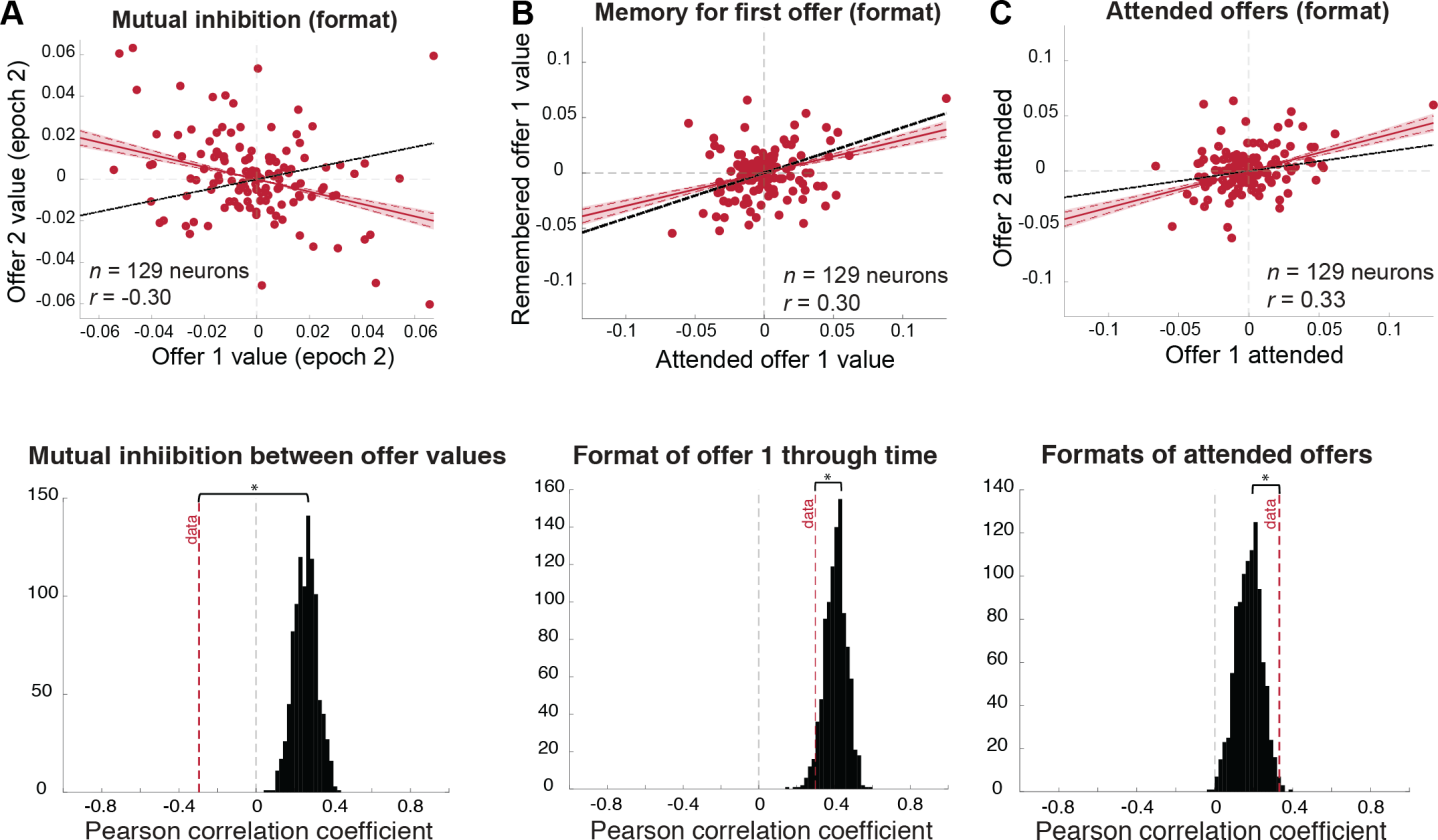
Formats of encoding values of the 2 offers support a 1-pool architecture of value-based choice. Red solid lines indicate mean correlation, red shaded area indicates 99% credible intervals, red dashed lines (in scatterplots) indicate 95% credible intervals, and black dashed lines indicate the correlation expected under a chance model. **(A)** Mutual inhibition during comparison: (top) scatterplot indicating the strength and direction of modulation in response to offer 1 and offer 2 (both during comparison) for each individual neuron in the population. The negative correlation indicates that neurons encode the values of the first and second offers in opposing formats during comparison. (Bottom) this correlation is significantly smaller than would be expected by chance (black distribution; permutation test with 1,000 permutations: *p* < 0.001). **(B)** Encoding of offer 1 value through time: (top) scatterplot indicating the strength and direction of modulation in response to offer 1 between the first and second epochs for each individual neuron in the population. The value of the first offer is encoded in correlated formats when it is attended (epoch 1) and when it is remembered (epoch 2). (Bottom) this correlation is not as strong as we would expect by chance (black dashed line; permutation test with 1,000 permutations: *p* = 0.038). This result holds when the outlier cell is removed. **(C)** Aligned encoding of attended offer values. (Top) scatterplot indicating the strength and direction of modulation in response to attended offers (offer 1 in the first epoch versus offer 2 in the second epoch). Values of attended offers are encoded in correlated formats across time, in contrast to 2-pool model predictions of mutual inhibition through time. (Bottom) this correlation is significantly larger than expected by chance (black distribution; permutation test with 1,000 permutations: *p* = 0.0060). This result holds when the outlier cell is removed. *Data used to generate these plots can be found at https://doi.org/10.5061/dryad.h52f8*.

A putative neuronal signature of value comparison via mutual inhibition is an anticorrelation between formats for 2 values [27,40,41]. Such an anticorrelation effectively subtracts them. The resulting signal can then be read out and its sign indicates the result of the comparison and thus the ensuing decision. We investigated the relationship between the encodings of the values of the 2 offers at the presumed time of comparison when the second offer appeared and the first was presumably stored in working memory. We found that these 2 formats are anticorrelated (Pearson correlation: *r* = −0.30, 99% credible interval: [−0.35, −0.23]; Fig 7A, top panel). This value is significantly less than would be expected by chance (permutation test: *p* < 0.0001; Fig 7A, bottom panel). The same analysis performed on the absolute regression coefficients (to examine only the strength of encoding, regardless of direction of modulation) suggests that overlapping populations of neurons respond to both offer values during the comparison stage (Pearson correlation of absolute regression coefficients: *r* = 0.42, 99% credible interval: [0.33, 0.50]).

The anticorrelation between offer value representations indicates that dACC does not encode the values of the offers independently but only encodes their values relative to each other. Further analysis indicates that the strengths with which offer values are encoded do not differ significantly (Wilcoxon signed rank test: *z* statistic = −0.730, *p* = 0.466). This finding suggests that neuronal activity in this region represents an unweighted difference signal, effectively subtracting the 2 offer values (rather than, say, partially normalizing the value of the second to the value of the first).

Correlates of mutual inhibition are consistent with both an underlying 2-pool architecture and with a 1-pool architecture. On one hand, 2 separate populations of neurons, each encoding one of the offer values, may be distinct, but each competitively inhibits the other population, leading to our observed anticorrelated formats (we will call this a 2-pool architecture). Because each neuron still responds to the values of both offers (either directly or via inhibition from the other population), our finding of overlapping populations does not falsify this model. On the other hand, these findings could indicate a single pool of neurons that respond to the offer values differently, effectively implementing value subtraction within each neuron (we will refer to this as a 1-pool architecture). In this model, no neurons are permanently dedicated to the representation of either offer value. To differentiate between these 2 proposed architectures, we take advantage of the asynchronous presentation of offers in our task to test 2 additional predictions: retention of offer 1 value and mutual inhibition over time.

#### Testing hypothesis 2: Offer 1 value is retained through time yet weakly influenced by offer 2 value

Under a 2-pool model, 1 pool of neurons is dedicated to encoding the value of the first offer through time. If this is true, we should see overlapping populations of neurons being activated in a similar fashion when the first offer is presented and when it is remembered, effectively “retaining” the value of the offer when it is no longer on the screen.

More specifically, we would expect to see a positive correlation between the encoding of the first offer’s value across the first epoch (when it is attended) and the second epoch (when it is no longer on the screen and must be remembered), because presumably the same population of neurons is permanently involved in encoding this offer’s value. We do, in fact, observe this positive correlation (Pearson correlation: *r* = 0.30, 99% credible interval: [0.24, 0.36]; Fig 7B, top panel). A population analysis also suggests that it is largely the same population of neurons encoding this value, rather than 2 separate populations (Pearson correlation of absolute regression coefficients: *r* = 0.48, 99% credible interval: [0.39, 0.56]). However, compared to a chance model, in which neurons encode the values of both offers but don’t differentiate between them, the format correlation is lower than would be expected (i.e., ceiling measure *r* = 0.41; permutation test: *p* = 0.038; Fig 7B, bottom panel; see Materials and methods for details). The population correlation is significantly stronger than we would expect by chance (*r* = 0.33; permutation test: *p* = 0.017). This is not due to levels of noise in our data, because our chance model analysis uses the exact same data, except shuffled, and detects a stronger correlation. Thus, while the value of the first offer is retained across epochs, its format is still modestly influenced by the appearance of offer 2. This evidence suggests that, while the value of the first offer is retained in the population, it is not retained in the same format for comparison with the second offer. While a 2-pool architecture posits that separate neuronal pools interact through mutual inhibition (as illustrated above), neurons in this framework should still respond similarly to the value of the first offer, faithfully retaining its value for comparison with the second offer.

#### Testing hypothesis 3: Offer values are not mutually inhibitory across time

If separate pools of neurons are dedicated to encoding each offer value, neurons excited by the offer 1 value in the first epoch should be inhibited by the offer 2 value in the second epoch, and vice versa. This prediction is an extension of the mutual inhibition prediction tested above, only through time, and is a prediction that follows from the 2-pool architecture hypothesis. We do not see any such pattern in our data. In fact, attended offers are encoded in strongly positively correlated formats (Pearson correlation: *r* = 0.33, 99% credible interval: [0.27, 0.39]; Fig 7C, top panel), more so than we would expect under a chance model (i.e., ceiling measure *r* = 0.17; permutation test: *p* = 0.006; Fig 7C, bottom panel). There is no separation in the populations activated in response to these offers, either; in fact, we see a significant overlap between the populations responding to offers 1 and 2 when they appear on the screen (Pearson correlation of absolute regression coefficients: *r* = 0.30, 99% credible interval: [0.22, 0.38]). This is not significantly different from a chance model (i.e., ceiling measure *r* = 0.22; permutation test: *p* = 0.13). Our data thus support a 1-pool hypothesis. Specifically, they suggest that the same population of neurons is activated in similar formats to offers being attended, regardless of offer identity.

## Discussion

We measured encoding of value in dACC neuron ensembles while monkeys performed a 2-option gambling task. Offers appeared in sequence, which let us observe choice occurring serially and let us experimentally control the focus of attention. We hypothesized that dACC neurons implement a comparison-to-reference mechanism in which the first offer serves at first as a reference to which the second is compared. Consistent with this hypothesis, neuronal encoding of the first offer was partially independent of the choice ultimately made, whereas neuronal encoding of the second offer was wholly dependent on the upcoming choice. We observed this discrepancy in decision dependence even when the encodings of offers’ values were analyzed within the same epoch, indicating a fundamental difference in how the first versus the second offer’s value was represented. The 2 offers were encoded by largely overlapping sets of neurons, consistent with the idea that comparison occurs within a single pool rather than in the form of competition between 2 discrete pools of neurons. These results suggest that economic choice can occur through comparison-to-reference mechanisms similar to those that apply to memory-guided decisions.

We developed new permutation analyses that allowed us to differentiate true ensemble coding differences from spurious differences due to noise. Surprisingly, ensemble responses to the first offer were partially dependent on the upcoming choice, meaning that the ensemble behaved qualitatively differently (not just gain changes, for example) depending on whether the offer would be chosen. This differentiation widened over time, as the similarity between value-encoding formats shrank. This finding indicates that responses to the first offer are partially post-decisional. They indicate that the neural response is not simply a representation of value but also includes responses associated with the specific choice to be made. Whether an internal commitment to the action has been made, we cannot say.

Several previous results highlight the post-decisional nature of value encodings in dACC [51,52]. Others indicate a direct role for this area in decision-making. Our results suggest a solution to these contradictory sets of results: the area does participate in decisions but because of the rapid nature of most studies, the post-decisional response is strongest. Indeed, our results suggest that the distinction between pre- and post-decisional is a misleading one. These results thus support a recent argument made by Hunt and colleagues [53]. In this study, Hunt et al. interpret the presence of decisional information as a correlate of decision formation, rather than an indicator of a strictly evaluative, post-decisional role.

Our results endorse the idea that value comparison in dACC occurs within a single pool of neurons, not between 2 discrete pools whose responses correspond to each option. The 2-pool model is one that is central to many models of value-based choice [25–29]. Critically, the sequential nature of our task allowed us to test for the first time a key prediction of 2-pool models. Such models make 2 predictions. First, a pool of neurons is hypothesized to maintain the value of the offer it represents through time. Our data do not support this prediction: the format of encoding the first offer’s value changes when the second offer appears, suggesting that this representation is not faithfully maintained for comparison with the second offer’s value. Second, 2-pool models predict that offer values attended sequentially will be encoded in opposing formats. Again, our data do not support this hypothesis: overlapping pools of neurons respond similarly to the values of both offers when each is attended. These findings support a mechanism of comparison in which neural activity is attentionally aligned, previously reported in the vmPFC and the VS [42,43]. Note that we do find mutual inhibition between offers when both have been viewed (i.e., in the second epoch, when offer 2 is attended and offer 1 is presumably held in working memory). This result corroborates findings from previous studies [27,40,41,50] but, as we show here, is not sufficient evidence for an underlying 2-pool architecture.

Our results must be interpreted in light of 2 caveats. First, because we present offers sequentially (in order to experimentally control which offer is attended at each point in time), we cannot fully distinguish between the effects of attention and those of order of presentation. Other paradigms relying on eye position, for example, could be used to determine the locus of attention at each point in time, although at least 1 previous study shows that overt fixation and covert attention are not always aligned [7]. Second, it is possible that endogenous fluctuations in attention or arousal influence choice and would thus appear as a post-decisional encoding of value (especially the value of the first offer). We believe this is a semantic difference rather than a scientific one: we believe the process of making a decision includes all factors contributing to a choice. These include factors that are relevant to the decision at hand—such as the values of the offers—as well as other variables—such as fluctuations in attention, arousal, etc.

While our results offer an explanation for serial choice processes, there is some reason to believe that a great deal of even ostensibly simultaneous choice actually reflects serial processing. First, our gaze (overt attention) and focus of attention (covert attention) are generally aligned and limited to 1 position [10,11]. Indeed, recent studies suggest overt or covert attention may structure the accumulation of evidence about different options [1,2,7] and emphasize the importance of the interaction between attentional effects and value on choice [8,54,55]. Second, foraging decisions, presumably a naturalistic form of decision-making, preferentially occur in a sequential context [6,56,57]. Nonetheless, it is an open question to what extent the comparison-to-reference mechanism we propose will generalize to other types of decisions.

A plethora of previous studies have implicated the dACC in the decision-making process. Kennerley and colleagues [31] find the strongest, most integrated value signals in dACC, and argue that this area is critical for value updating and subsequent value-based decision-making [58]. This region is also known for representing quantities in a manner that is context dependent, for example, relative to the values of other available options [59] or relative to their order in a sequence [60,61]. This region is also known for its role in directing attention to valuable and salient stimuli [62]. However, while the importance of the dACC in choice seems evident, its role in this process remains a subject of debate [38,63,64]. Most relevant to the present study, one view holds that the dACC is a critical site of value-based comparison in choice and is thus pre- or mid-decisional (depending on how one defines these terms [34,39,65]). Another school holds that the dACC can be situated largely post-decisionally, meaning that it receives the outputs of decisions and uses this information to monitor or adjust future behavior [51,52]. This post-decisional role in turn is consistent with a literature emphasizing a largely monitoring role of the dACC [38,66]. Our results here suggest the 2 views can be reconciled: the first offer sets the context of comparison (including pre-decisional aspects), thus allowing the second offer to encode the outcome of the decision (post-decisional aspects). In other words, our findings push against the dichotomy between pre- and post-decisional processes, arguing that this region is involved in decision formation itself. More broadly, it is possible that the dACC and even the brain more generally does not represent value divorced of upcoming decisions and plans of action (related arguments have previously been made [67,68]).

Our findings fit particularly well with a recent study by Boorman and colleagues [4]. There, the authors find that dACC activity during choice correlates well with the value of the currently available option relative to a default option with the highest long-term value. We extend these findings by showing that dACC activity at the single-unit level also reflects this default/alternative framework of comparison and in contexts in which the default is defined by order of presentation, rather than by long-term value. A recent imaging study by Lopez-Persem and colleagues [16] also argues for this mechanism, and finds correlates of it in the vmPFC. Although they find no correlates of default-to-alternative evaluation in the dACC, we posit that this may be due to the relative weakness of these decision-dependent signals in the dACC, which may make them difficult to detect in aggregate measures such as BOLD. This finding would fit into a more general framework in which different brain regions along the processing stream transform value information into decisions and information necessary for post-decisional, evaluative processes. We expect this transformation to be gradual, such that “pre”- and “post”-decisional correlates would be observed in multiple brain regions in varying degrees. Further research will be required to test this theory and understand how single-neuron correlates we observe in our study would translate into aggregate measures, such as BOLD.

More generally, our findings suggest that dACC neurons carry value signals that are not abstract representations of value—or even necessarily consistent across contexts within a single task (similar patterns of neural activity have been observed in the orbitofrontal cortex [69]). These ideas in turn raise the possibility of a subtle but important shift in the interpretation of neural correlates of value. A representational approach views such neural correlates as a representation of an abstract value signal, that is, a signal whose function is the same as its form—to convey to downstream structures the abstract value of the option, regardless of the computation this quantity is involved in. Instead, our results support a functionalist interpretation—value correlates may have diverse purposes that, while they correlate with value, are not strictly representational (this argument has been previously expounded in more detail [70]). Beyond representing an abstract quantity to be read out by a downstream brain region, these signals function to enable mental processes that rely on option value and may thus serve different functions depending on the eventual decision [71].

## Materials and methods

### Ethics statement

All procedures were approved by the University Committee on Animal Resources at the University of Rochester and were designed and conducted in compliance with the Public Health Service’s Guide for the Care and Use of Animals (protocol UCAR-2010-169). Two male rhesus macaques (*Macaca mulatta*, subject B: age, 8 years, 11 months; subject J: age, 10 years, 9 months) served as subjects. A small prosthesis for holding the head was used. Animals were habituated to laboratory conditions and then trained to perform oculomotor tasks for liquid reward. A Cilux recording chamber (Crist Instruments) was placed over the dACC. Position was verified by magnetic resonance imaging with the aid of a Brainsight system (Rogue Research Inc.). Animals received appropriate analgesics and antibiotics after all procedures. Throughout both behavioral and physiological recording sessions, the chamber was kept sterile with regular antibiotic washes and sealed with sterile caps. All recordings were performed during the animals’ light cycle, between 8 AM and 5 PM.

Some of the data for dACC recordings were previously published [42]; all analyses presented here are new.

### Recording site

We approached the dACC through a standard recording grid (Crist Instruments). We defined the dACC according to the Paxinos atlas [72]. Roughly, we recorded from a region of interest lying within the coronal planes situated between 29.50 and 34.50 mm rostral to interaural plane, the horizontal planes situated between 4.12 and 7.52 mm from the brain’s dorsal surface, and the sagittal planes situated between 0 and 5.24 mm from medial wall. The atlas called these Areas 8/32 and 9/32; we prefer to call them Area 24 [38]. Our recordings were made from a central region within this zone. We confirmed recording location before each recording session using our Brainsight system with structural magnetic resonance images taken before the experiment. Neuroimaging was performed at the Rochester Center for Brain Imaging on a Siemens 3T MAGNETOM Trio Tim using 0.5-mm voxels. We confirmed recording locations by listening for characteristic sounds of white and gray matter during recording, which in all cases matched the loci indicated by the Brainsight system.

### Electrophysiological techniques, eye tracking, and reward delivery

All methods used were described in previous manuscripts [40] and largely reproduced here. Single electrodes (Frederick Haer & Co., impedance range 0.8–4 MU) were lowered using a microdrive (NAN Instruments) until waveforms of between 1 and 3 neuron(s) were isolated. Individual action potentials were isolated on a Plexon system (Plexon, Inc.). Neurons were selected for study solely on the basis of the quality of isolation; we never preselected based on task-related response properties. All collected neurons for which we managed to obtain at least 250 trials were analyzed.

Eye position was sampled at 1,000 Hz by an infrared eye-monitoring camera system (SR Research). Stimuli were controlled by a computer running Matlab (Mathworks) with Psychtoolbox [73] and Eyelink Toolbox [74]. Visual stimuli were colored rectangles on a computer monitor placed 57 cm from the animal and centered on its eyes (Fig 2A). A standard solenoid valve controlled the duration of juice delivery. The relationship between solenoid open time and juice volume was established and confirmed before, during, and after recording.

### Behavioral task

Monkeys performed a 2-option gambling task. The task was similar to one we have used previously [40,41,75], with 2 major differences: first, monkeys gambled for virtual tokens rather than liquid rewards, and second, outcomes could be losses as well as wins.

Two offers were presented on each trial. Each offer was represented by a rectangle 300 pixels tall and 80 pixels wide (11.35° of the visual angle tall and 4.08° of the visual angle wide). Twenty percent of options were safe (100% probability of either 0 or 1 token), while the remaining 80% were gambles. Safe offers were entirely red (0 tokens) or blue (1 token). The size of each portion indicated the probability of the respective reward. Each gamble rectangle was divided horizontally into a top and bottom portion, each colored according to the token reward offered. Gamble offers were thus defined by 3 parameters: 2 possible token outcomes, and the probability of the top outcome (the probability of the bottom was strictly determined by the probability of the top). The top outcome was 10%, 30%, 50%, 70%, or 90% likely.

Six initially unfilled circles arranged horizontally at the bottom of the screen indicated the number of tokens to be collected before the subject obtained a liquid reward. These circles were filled appropriately at the end of each trial, according to the outcome of that trial. When 6 or more tokens were collected, the tokens were covered with a solid rectangle while a liquid reward was delivered. Tokens beyond 6 did not carry over nor could number of tokens fall below zero.

On each trial, one offer appeared on the left side of the screen and the other appeared on the right. Offers were separated from the fixation point by 550 pixels (27.53° of the visual angle). The side of the first offer (left and right) was randomized by trial. Each offer appeared for 600 ms and was followed by a 150-ms blank period. Monkeys were free to fixate upon the offers when they appeared (and in our observations almost always did so). After the offers were presented separately, a central fixation spot appeared and the monkey fixated on it for 100 ms. Following this, both offers appeared simultaneously and the animal indicated its choice by shifting gaze to its preferred offer and maintaining fixation on it for 200 ms. Failure to maintain gaze for 200 ms did not lead to the end of the trial but instead returned the monkey to a choice state; thus, monkeys were free to change their mind if they did so within 200 ms (although in our observations, they seldom did so). A successful 200-ms fixation was followed by a 750-ms delay, after which, the gamble was resolved and a small reward (100 μL) was delivered—regardless of the outcome of the gamble—to sustain motivation. This small reward was delivered within a 300-ms window. If 6 tokens were collected, a delay of 500 ms was followed by a large liquid reward (300 μL) within a 300-ms window, followed by a random intertrial interval (ITI) between 0.5 and 1.5 s. If 6 tokens were not collected, subjects proceeded immediately to the ITI.

Each gamble included at least one positive or zero outcome. This decreased the number of trivial choices presented to subjects and maintained motivation.

### Statistical methods for behavior

Subjective values for each gamble were estimated based on subjects’ behavior performance in each test session, according to the following formula:

$$SV=p*win^{\alpha }+(1-p)*loss^{\beta }$$

(the same approach used by Yamada and colleagues [76]). Because our task includes both wins and losses, we fit a parameter α for wins and another parameter β for losses. A value for α greater than 1 and a value for β less than 1 both indicate risk seeking.

Both subjects were risk seeking on average (values of α > 1 or β < 1 both indicate risk seeking; subject B: average α = 1.21, SD = 0.409, average β = 0.0764, SD = 0.132; subject J: average α = 1.60, SD = 0.404, average β = 0.0216, SD = 0.0530).

We also fit subjective values conditioned on the number of tokens the subject had accumulated as of the beginning of each trial. We did this by fitting the above equation to trials in each possible tokens-accumulated condition (accumulated tokens = 0, 1, …, 5). We thus obtained 6 different parameters for gains and losses for each subject (S2 Table).

We used the subjective values fit by the parameters obtained using these 2 methods to replicate our analyses of significantly-modulated neurons (S3 Table) and beta correlation analyses (S4 Table).

We fit logistic regression models of behavior to predict choice of the first versus second offer. To ensure that subjects did, in fact, pay attention to both offers, we fit a model in which the values of the first and second offers were the predictors of interest, while also including the number of tokens already accumulated, the side the first offer appears on, and the choice eventually made to explain any variance these variables might contribute to. To determine whether subjects pay attention to all features of an offer, we use an extended model with the 3 variables characterizing each offer (the 2 possible outcomes and the probability of the larger outcome) included as predictors, controlling for the same variables mentioned above. We fit such a model for each behavioral session and obtained the regression weights associated with each of the variables of interest. We then tested the vector of these variables across all sessions using a 1-sample *t* test to determine whether they differ significantly from zero.

### Statistical methods for physiology

Peristimulus time histograms (PSTHs) were constructed by aligning spike rasters to the presentation of the first offer and averaging firing rates across multiple trials. Firing rates were calculated in 20-ms bins but were generally analyzed in longer (500-ms) epochs. This method is standard in our lab and was described in a previous manuscript [40].

Firing rates were normalized by subtracting the mean and dividing by the standard deviation of the entire neuron’s PSTH. We tested for significant neuronal modulation using a multiple linear regression, including the following task-relevant variables: expected/subjective value of offers 1 and 2, the number of tokens collected as of the beginning of the trial, the side the first offer appeared on, and the side of the chosen offer.

Analysis epochs were chosen a priori before data analysis began to reduce the likelihood of *p*-hacking. The first and second offer epochs were defined as the 500-ms epoch beginning 100 ms after the offer was presented, to account for information-processing time. These epochs were used in previous studies of choice behavior [40,41]. All fractions of modulated neurons were tested for significance using a 2-sided binomial test. All binomial tests throughout the manuscript were 2-sided.

### Format and population correlation analyses

We used beta correlation analyses to assess whether neurons represented 2 variables (or the same variable at different time periods) using similar/orthogonal/opposing formats, in overlapping/orthogonal/distinct populations. To do this, we first found the regression coefficient associated with the variable of interest (controlling for other task variables, listed above) per neuron. We then combined these regression coefficients into a vector of the same length as the number of neurons in our sample. This vector indicates the strength and direction of modulation for each individual neuron in the population, in response to a particular variable in a particular epoch. We call this the population “format.” We compared different formats by finding the Pearson correlation coefficient between them.

We modify this method slightly to account for the noise levels of each individual neuron’s encoding of each variable of interest, which our existing method cannot account for. We used a Bayesian regression to obtain a probabilistic distribution over each regression coefficient for each neuron, rather than an individual value per neuron. We sampled 10,000 regression coefficients from this distribution per neuron to obtain 10,000 potential formats for the population. We then performed the correlation analyses on each of these samples, thus generating a distribution of 10,000 correlation coefficients. This is a more robust estimate of the correlation between formats, as it takes into account the uncertainty inherent in estimating any individual regression coefficient and allows us to view the spread of the distribution of this correlation when this significant source of noise is taken into account. Credible intervals of 99% allowed us to estimate the likely range of the correlation coefficients with 99% certainty.

The Pearson correlation coefficient between signed regression coefficients indicates whether variables were represented in a similar format, i.e., directionality of tuning across the population. A positive correlation indicates a preservation of directionality, while a negative correlation suggests variables were represented in opposing directionality of firing rate modulation. No correlation suggests orthogonal formats.

Similarly, the Pearson correlation coefficient between unsigned regression coefficients indicates whether similar neuronal populations tended to be involved in encoding the 2 variables in question. A positive correlation indicates overlapping populations, while a negative correlation indicates separate ones. A lack of correlation suggests orthogonal populations (i.e., encoding 1 variable does not affect the neuron’s likelihood of encoding the other variable).

We then compare these distributions of correlation coefficients to distributions that would be obtained under a chance model. For the first set of analyses, meant to differentiate between the 1-pool and 2-pool hypotheses, we assume a chance model is one in which, during the second epoch, neurons encode the values of both offers but don’t differentiate between them. We achieve this by shuffling the values of the 2 offers and using these new shuffled vectors as predictors in our regression model. For the second set of analyses, examining the encodings of accepted and rejected offers, we assume a chance model in which neurons do not differentiate between values according to whether they will later be accepted or rejected. To achieve this, we shuffle trials across these 2 categories (offer 1 or 2 accepted/rejected) at random. These chance models achieve a permutation of the existing data, which we then use for the same beta correlation analyses explained above. We then compare the mean correlation of our actual data to the distribution of mean correlations obtained from each of 1,000 permutations of our data. This form of test allows us to (1) ensure that we have enough signal in our data to detect significant correlations, and (2) determine whether the variable of interest does, in fact, play a role in differentiating the formats/populations involved. As we have often found in this study, a positive correlation between formats, while in itself informative, may not actually be as strong as would be expected purely by chance given this dataset, which is also, in itself, an important finding that informs our theories and interpretations.

Data were deposited in the Dryad depository http://dx.doi.org/10.5061/dryad.h52f8 [77].

## Supporting information

S1 Text Spatial bias is not influenced by trial difficulty. (DOCX)

S2 Text Replicating neural analyses with subjective offer values. (DOCX)

S3 Text A closer look at pre-decisional versus post-decisional encoding within the value-tuned neuronal population. (DOCX)

S4 Text Visualizing influence of upcoming choice on early trial activity using principal component analysis. (DOCX)

S5 Text Influence of decision on value encoding in the vmPFC and VS. vmPFC, ventromedial prefrontal cortex; VS, ventral striatum.
(DOCX)

S1 Table Subjects rely on all option values to make a choice. We use a logistic regression model, including the variables listed in the table below, controlling for number of tokens the subject has as of the beginning of the trial, the side the first offer appeared on, and the chosen side. We use these variables to predict whether the subject chose the first offer presented. If significant, coefficients for the first offer should be positively skewed, while coefficients for the second offer should be negatively skewed. We perform this analysis on each behavioral session, then test whether the resulting regression coefficients differ significantly from zero (related to behavioral analyses in the main text).
(XLSX)

S2 Table Percent of neurons modulated in response to different task variables (related to Fig 2 and single-neuron modulation results in the main text). (XLSX)

S3 Table Biases in modulation in significant population and across the entire population (related to single-neuron modulation results in the main text). (XLSX)

S4 Table Beta-correlation results for format and population analyses, when offers are accepted versus rejected (related to Figs 3, 4 and 5 in the main text). (XLSX)

S5 Table Beta-correlation results for format and population analyses, for 1-pool model results (related to Fig 7 in main text). (XLSX)

S1 Fig Visualizing the first PC when offers are later accepted/rejected. The first PC shows a general rise in activity, culminating after both offers have been shown and the subject has presumably made a choice. Even this first PC shows significant differences between trials in which the first versus the second offer is ultimately chosen. Activity peaks significantly when the ultimately chosen offer appears, and overall activity peaks at a higher level when offer 1 is chosen rather than offer 2. PC, principal component.
(TIF)

S2 Fig Visualizing the second and third PCs when offers are later accepted/rejected. The second and third PCs also show earlier peaks in activity when offer 1 (versus 2) is chosen, which are more pronounced than those observed in the first PC. No significant differences were observed in PCs 4 and 5. These findings additionally support our general hypothesis, in which offer value and choice signals are intimately intertwined, and both affect firing rates early on in the trial. PC, principal component.
(TIF)

S3 Fig Correlations between offer formats when they are later accepted/rejected, visualized through time. Solid line shows the mean correlation between accepted and rejected regression coefficients for each offer, aligned to offer onset. Shading indicates the upper and lower bounds of the 99% credible interval. The first vertical line indicates the appearance of the first offer. The second vertical line indicates the appearance of the second offer. The third vertical line indicates the disappearance of the second offer and the end of the second epoch, after which the subject would be allowed to indicate his choice. These plots reiterate and emphasize our results. Offer 1 encodings across decision contexts start out fairly correlated, and this correlation decreases as the trial proceeds and information about the second offer appears. Offer 2 appears to be encoded in less correlated formats than offer 1 when it appears. Results in the main text show that these correlations, although mostly positive, are less than would be expected from randomly permuted trials.
(TIF)

S4 Fig Correlations between first and second offer formats, visualized through time. The solid line shows the mean correlation between offer 1 and offer 2 values, aligned to the offer 2 onset. Shading indicates the upper and lower bounds of the 99% credible interval. The first vertical line indicates the appearance of the second offer. The second vertical line indicates the end of the offer 2 epoch, after which the second offer disappears. This plot also confirms our findings in the main text. It also shows that the mutual inhibition signal fades and offers are no longer encoded in opposing formats as the trial transitions into the choice epoch.
(TIF)

## Abbreviations

dACC: dorsal anterior cingulate cortex
KS: Kolmogorov-Smirnov
vmPFC: ventromedial prefrontal cortex
VS: ventral striatum
