# Supplementary material for: Correlates of decisional dynamics in the dorsal anterior cingulate cortex
Source: PLoS Biol. 2017 Nov 15;15(11):e2003091. doi: 10.1371/journal.pbio.2003091 (PMC5706721; doi:10.1371/journal.pbio.2003091)
Supplement: S1 Text — (DOCX) [file pbio.2003091.s001.docx]

We re-analyzed our behavior data using a logistic regression analysis, where we predicted whether the subject chose the offer on the left using the following behavioral variables: expected values of the left and right offers, the number of tokens accumulated as of the beginning of the trial, which offer was shown on the left (first or second) and, critically, trial difficulty (i.e. the absolute difference between the expected values of the left and right offers). We performed this analysis on each behavioral session’s data, and examined the regression coefficient associated with difficulty. The difficulty coefficient was never a significant predictor of chosen side for subject B (n = 0/26 behavioral sessions), and only predictive of subject J’s performance on 2 out of 39 behavioral sessions, which is not significant according to a two-sided binomial test (p = 1).
