# Supplementary material for: Correlates of decisional dynamics in the dorsal anterior cingulate cortex
Source: PLoS Biol. 2017 Nov 15;15(11):e2003091. doi: 10.1371/journal.pbio.2003091 (PMC5706721; doi:10.1371/journal.pbio.2003091)
Supplement: S2 Text — (DOCX) [file pbio.2003091.s002.docx]

In tables S2-S5, we replicate all the main analyses of our paper using subjective, instead of expected, values. Both measures provide a reasonable account of the monkeys' choices. Subjective values provide a more accurate one (and must do so, since it is fit to the data), although expected values have fewer assumptions. The relationship between the two is, by definition, a monotonic transform. Because of this, and because our analyses are all fundamentally linear regressions, the decision between use of subjective values vs. expected values does not qualitatively affect the results; it only modifies our ability to detect signal in the data. In any case, we favored expected values in the main text because they are the more statistically conservative measure.
