# Supplementary material for: Correlates of decisional dynamics in the dorsal anterior cingulate cortex
Source: PLoS Biol. 2017 Nov 15;15(11):e2003091. doi: 10.1371/journal.pbio.2003091 (PMC5706721; doi:10.1371/journal.pbio.2003091)
Supplement: S3 Text — (DOCX) [file pbio.2003091.s003.docx]

We state in our manuscript that value-encoding contains pre- as well as post-decisional elements: While value formats across choice conditions (accepted vs. rejected) are correlated less than would be expected by chance (indicating the presence of decisional information), they are still positively correlated (indicating a pre-decisional component). The following analysis allows us to further elaborate this statement, by examining the interaction between value-encoding and upcoming choice for every individual neuron significantly modulated by value.

We use stepwise linear regression models to determine which variables to keep in a model that best explains each individual neuron’s firing rate. We allow the analysis to fit any combination of the following variables, along with any of their interactions: offer values and side of chosen offer (the two variables of interest), as well as the side the first offer appears on, and number of tokens accumulated as of the beginning of the trial (control variables). We find that a significant proportion of neurons are tuned to offer values according to this analysis (epoch 1, offer 1: n = 62/129 neurons i.e. 48.1%; epoch 2, offer 1: n = 56/129 neurons i.e. 43.4%; epoch 2, offer 2: 54/129 neurons ie.. 41.9%). We also find a significant fraction of neurons with a significant interaction term between offer value and whether that offer was chosen (all p-values obtained using a two-sided binomial test: epoch 1, offer 1: n = 24/62 i.e. 38.7%, p = 8.8818 x 10^-16^; epoch 2, offer 1: n = 17/56 i.e. 30.4%, p = 1.1402 x 10^-9^; epoch 2, offer 2: n = 21/54 i.e. 38.9%, p = 4.9516 x 10^-14^). These proportions are also significant in the entire population (epoch 1, offer 1: n = 24/129 i.e. 18.6%, p = 2.5896 x 10^-8^; epoch 2, offer 1: n = 17/129 i.e. 13.2%, p = 2.5380 x 10^-8^; epoch 2, offer 2: n = 21/129 i.e. 16.3%, p = 1.8453 x 10^-6^).

Thus far, these results indicate that value encoding changes *somehow* depending on whether or not an offer is later chosen: it does not indicate whether these changes are changes of format (i.e. direction of firing-rate modulation), or simply changes in multiplicative gain. Still, one can detect a change of format if the following two conditions are satisfied: a) value coefficients and interaction term coefficients are negatively correlated, and b) interaction term coefficients tend to be larger than value coefficients. To understand why this is the case, it helps to examine (a minimal form of) the linear model fit by this method (we omit variables that don’t depend on value for ease of explanation):

$FR=\beta_{v}v+ \beta_{v*c}cv$

where *v* is the value of the offer and *c* is a binary variable indicating whether the offer was chosen. The second term of the equation constitutes the interaction term. Since *c* is a binary variable (equal to zero unless the offer is chosen), the interaction term only influences value-specific modulation if the offer is chosen. If this interaction term exceeds the value term in magnitude (i.e. abs(β_v*c_) > abs(β_v_)) but is opposite in sign, this effects a change in the direction of modulation of firing rates in response to value for this neuron (i.e. a change in format).

We tested this assumption within the population of neurons that exhibited a significant interaction between value and whether an offer was chosen (24 neuron for offer 1 in epoch 1, 17 neurons for offer 1 in epoch 2, and 21 neurons for offer 2 in epoch 2). Despite these small sample values, we were able to observe significant results in 2 out of 3 cases, aligned with a change in format *within this population*. The coefficients of the value and interaction terms for these neurons tended to be negatively correlated (satisfying the first condition), although not significantly so in the first epoch (Pearson correlation: epoch 1, offer 1: r = -0.21, p = 0.32; epoch 2, offer 1: r = -0.75, p = 0.0005; epoch 2, offer 2: r = -0.56, p = 0.0085). Moreover, interaction coefficients tended to be larger than value coefficients (satisfying the second condition; Wilcoxon signed-rank test: epoch 1, offer 1: Z-value = -3.57, p = 0.0004; epoch 2, offer 1: Z-value = -3.53, p = 0.0004; epoch 2, offer 2: Z-value = -3.91, p = 0.0001). These results thus illustrate more closely the post-decisional component of value-encoding in the significantly value-tuned population of dACC neurons in our data. However, we remind the reader that only 30-40% of value-tuned neurons exhibited this decision-dependent modulation of value-encoding (see exact numbers above); the remaining neurons did not exhibit any significant interaction between an offer’s value and whether it was later chosen. These neurons thus illustrate the pre-decisional portion of value-encoding in significantly value-tuned neurons.
