# Supplementary material for: Correlates of decisional dynamics in the dorsal anterior cingulate cortex
Source: PLoS Biol. 2017 Nov 15;15(11):e2003091. doi: 10.1371/journal.pbio.2003091 (PMC5706721; doi:10.1371/journal.pbio.2003091)
Supplement: S4 Text — (DOCX) [file pbio.2003091.s004.docx]

We sought to visualize the evolution of firing rates on trials when the first offer was chosen and trials when the second option was chosen. To achieve this, we use a standard dimensionality reduction method, Principal Component Analysis (PCA) on the average firing rate of each neuron on trials where offer 1 vs. 2 was chosen. A sliding 500 ms time window was used to characterize activity at each time-point, starting 1 second before the first offer appears, until 1 second after the second offer disappears. Below are plots illustrating the first 3 PC’s, which collectively account for 72.98% of the variance in our data. Data are aligned to the appearance of the first offer. Error shading illustrates the 95% confidence interval on each PC, computed using a permutation test (trials were shuffled at random and PCA analysis was repeated on this shuffled data. This procedure was repeated 100 times).
