# Supplementary material for: Correlates of decisional dynamics in the dorsal anterior cingulate cortex
Source: PLoS Biol. 2017 Nov 15;15(11):e2003091. doi: 10.1371/journal.pbio.2003091 (PMC5706721; doi:10.1371/journal.pbio.2003091)
Supplement: S5 Text — vmPFC, ventromedial prefrontal cortex; VS, ventral striatum. (DOCX) [file pbio.2003091.s005.docx]

We examined the influence of the decision on the encoding of offer value in two other brain regions: the ventromedial prefrontal cortex (vmPFC) and the ventral striatum (VS). Data from these regions were recorded in a slightly different gambling task (details in Strait et al. 2014 and Strait et al. 2015). These data show qualitatively similar (thought not identical) results as those from the dACC reported in this study, supporting a possible distributed neural architecture underlying value-based decisions.

Like in dACC, neurons in the **ventromedial prefrontal cortex (vmPFC)** show decision-dependent encoding of offer value for offer 1 in the first epoch (Pearson correlation coefficient r = 0.101, 99% confidence interval: [0.0310, 0.179]), differing significantly from what we would expect purely due to noise (i.e. ceiling measure obtained from permuted data: r = 0.255; p = 0.0130). We also find decision-dependent encoding of offer 2 during the second epoch (r = 0.133 [0.0582, 0.209], ceiling r = 0.239; p = 0.0360). However, unlike in dACC, we see no evidence of such decision-dependence for offer 1 encoding in the second epoch (r = 0.132 [0.0479, 0.205], ceiling r = 0.172; p = 0.25).

Neurons in the **ventral striatum (VS)** show decision-dependent encoding of both offers in both epochs, similar to neurons in dACC. Encoding of offer 1 in the first epoch depends on the upcoming decision, as indicated by a permutation test (r = 0.350 [0.280, 0.419], ceiling r = 0.559; p < 0.0001). Results are similar for offer 1 in the second epoch (r = 0.0864 [-0.0277, 0.2015], ceiling r = 0.22; p = 0.0130), as well as for offer 2 in this same epoch (r = 0.345 [0.267, 0.412], ceiling r = 0.654; p < 0.0001).
