# Supplementary figures and images for: Correlates of decisional dynamics in the dorsal anterior cingulate cortex
Source: PLoS Biol. 2017 Nov 15;15(11):e2003091. doi: 10.1371/journal.pbio.2003091 (PMC5706721; doi:10.1371/journal.pbio.2003091)

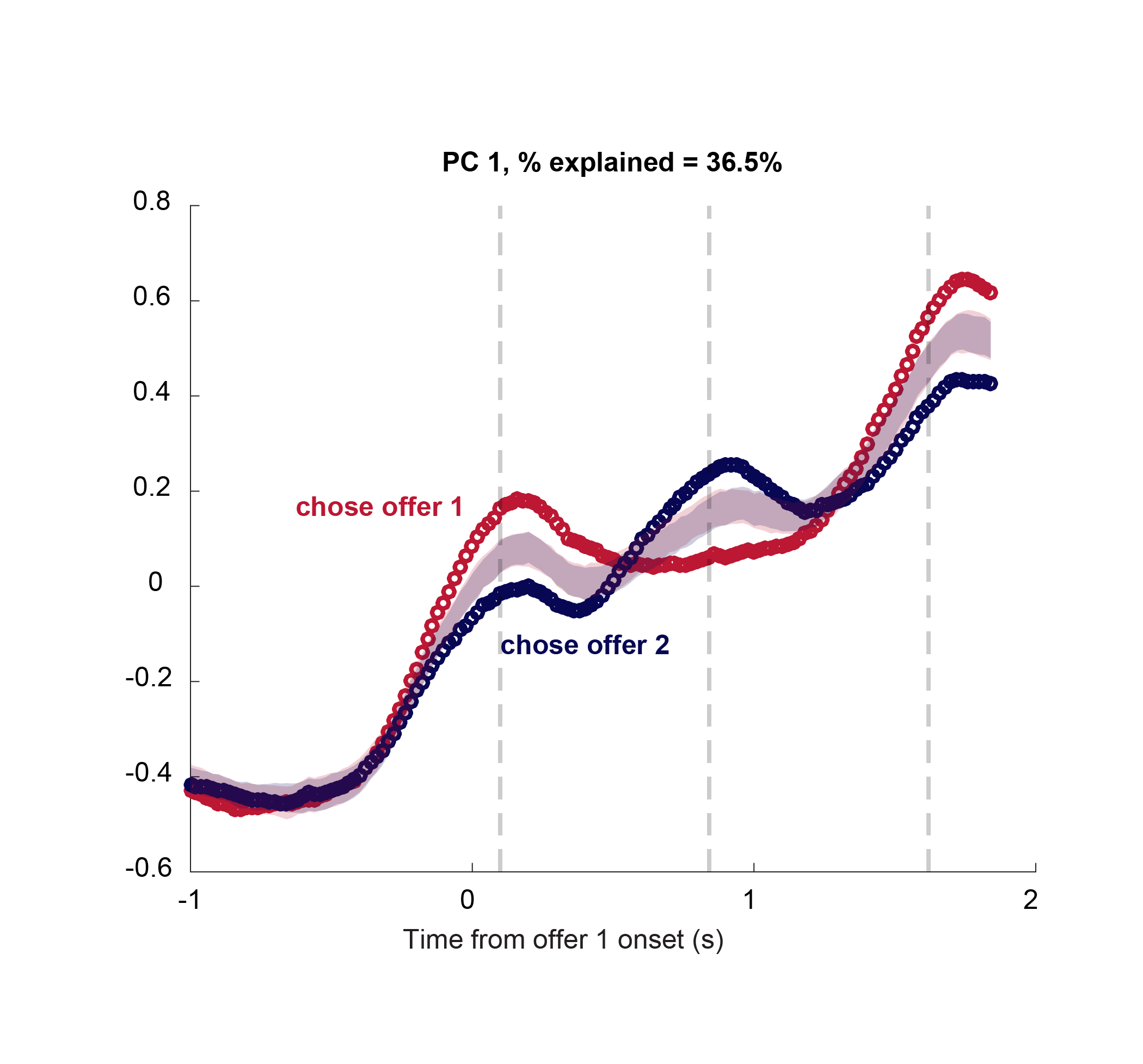

Supplement: S1 Fig — The first PC shows a general rise in activity, culminating after both offers have been shown and the subject has presumably made a choice. Even this first PC shows significant differences between trials in which the first versus the second offer is ultimately chosen. Activity peaks significantly when the ultimately chosen offer appears, and overall activity peaks at a higher level when offer 1 is chosen rather than offer 2. PC, principal component. (TIF) [file pbio.2003091.s011.tif]

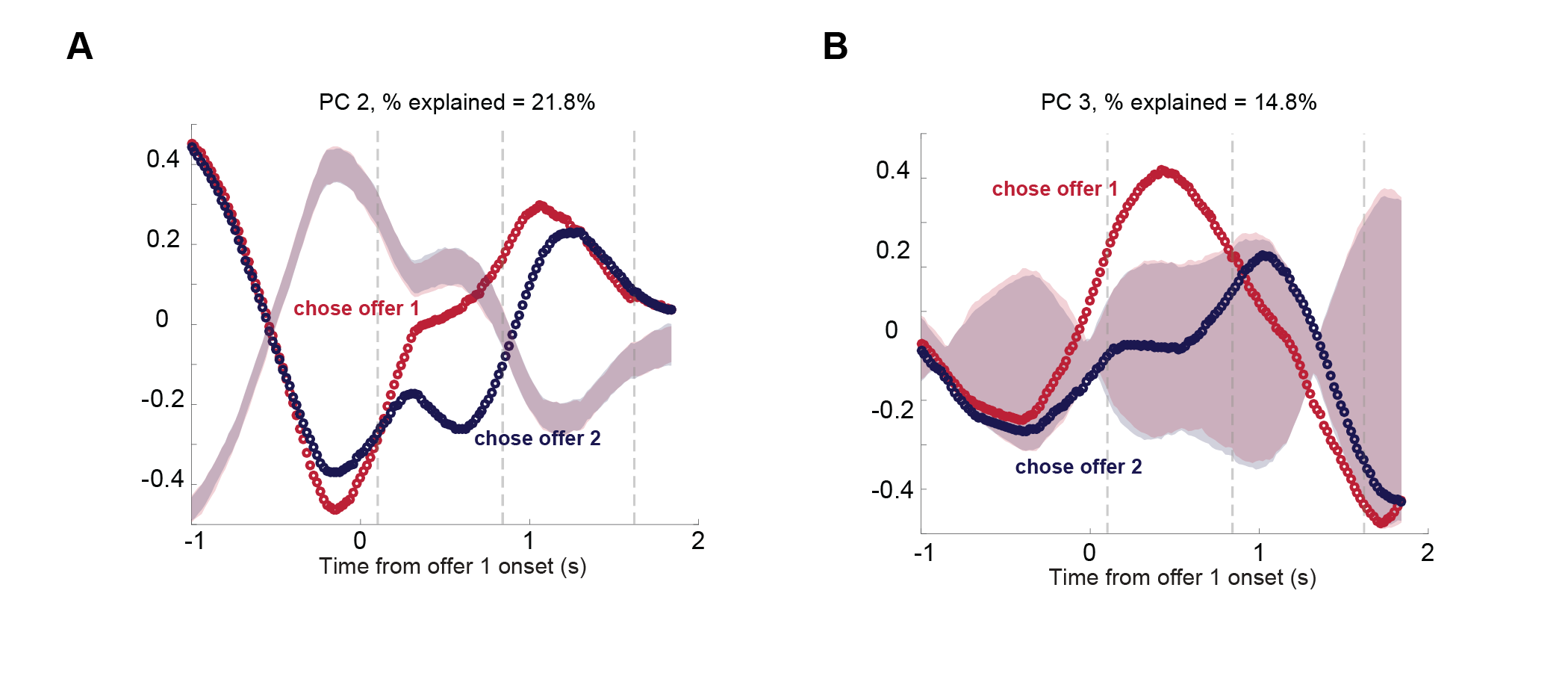

Supplement: S2 Fig — The second and third PCs also show earlier peaks in activity when offer 1 (versus 2) is chosen, which are more pronounced than those observed in the first PC. No significant differences were observed in PCs 4 and 5. These findings additionally support our general hypothesis, in which offer value and choice signals are intimately intertwined, and both affect firing rates early on in the trial. PC, principal component. (TIF) [file pbio.2003091.s012.tif]

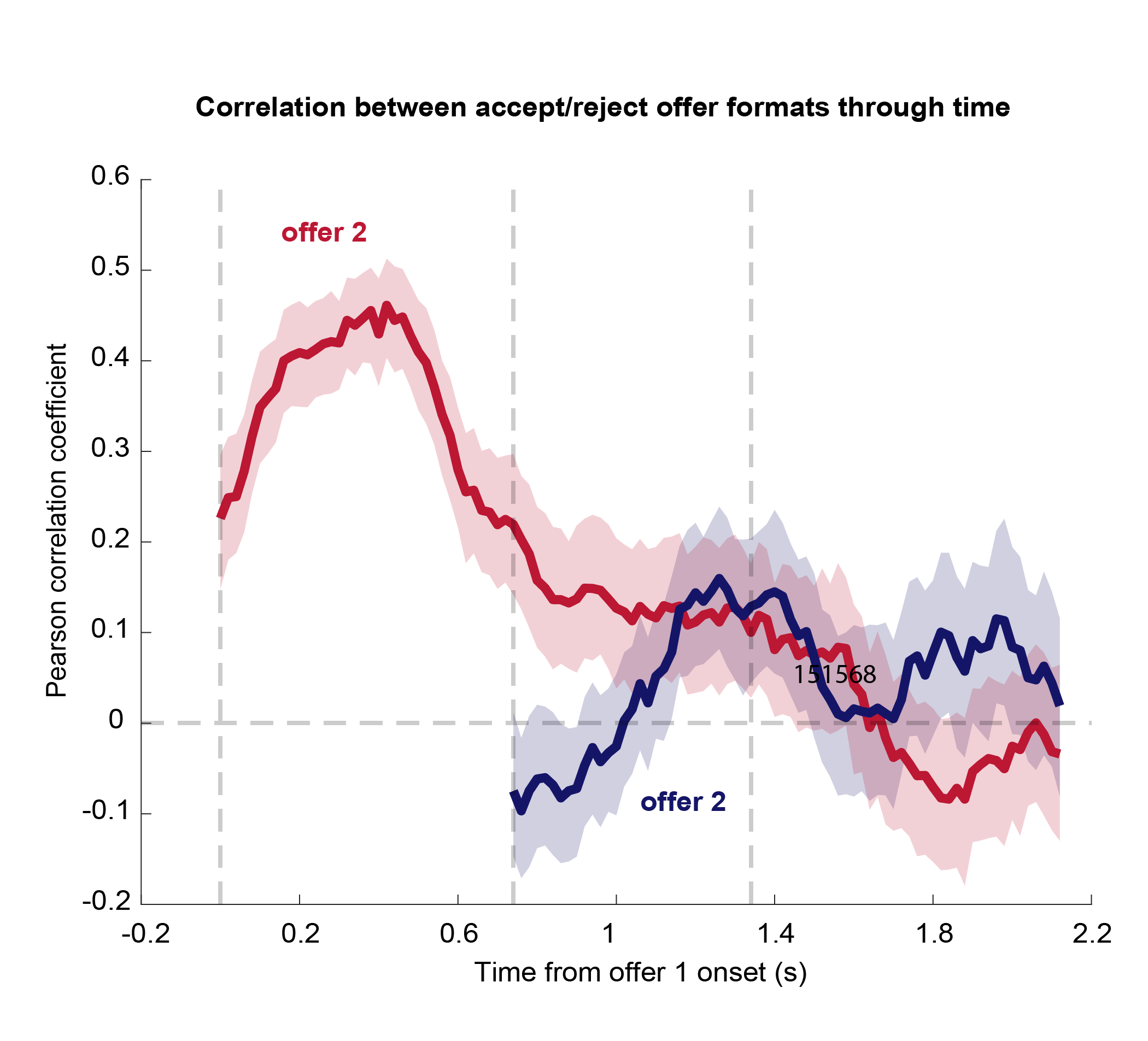

Supplement: S3 Fig — Solid line shows the mean correlation between accepted and rejected regression coefficients for each offer, aligned to offer onset. Shading indicates the upper and lower bounds of the 99% credible interval. The first vertical line indicates the appearance of the first offer. The second vertical line indicates the appearance of the second offer. The third vertical line indicates the disappearance of the second offer and the end of the second epoch, after which the subject would be allowed to indicate his choice. These plots reiterate and emphasize our results. Offer 1 encodings across decision contexts start out fairly correlated, and this correlation decreases as the trial proceeds and information about the second offer appears. Offer 2 appears to be encoded in less correlated formats than offer 1 when it appears. Results in the main text show that these correlations, although mostly positive, are less than would be expected from randomly permuted trials. (TIF) [file pbio.2003091.s013.tif]

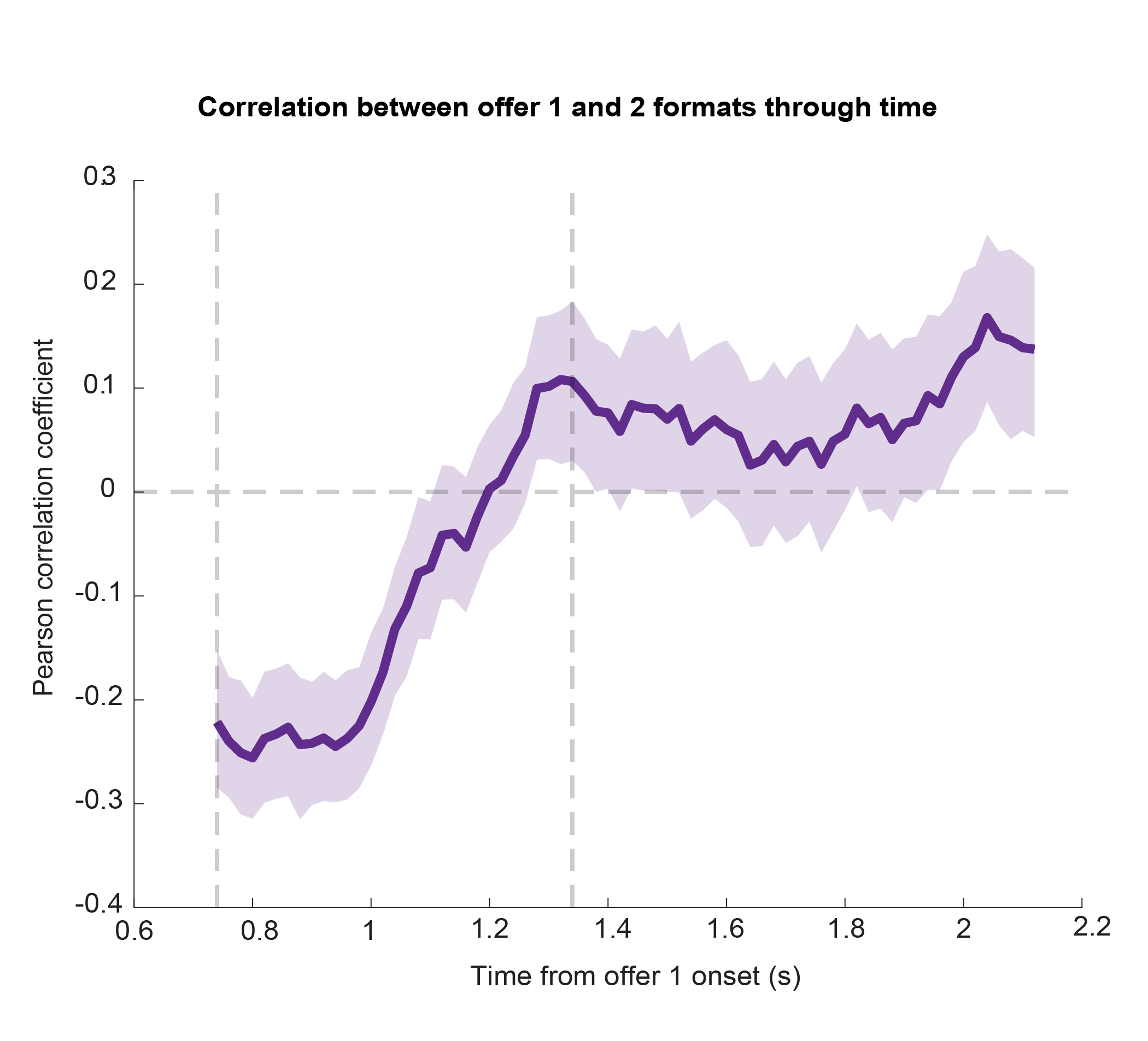

Supplement: S4 Fig — The solid line shows the mean correlation between offer 1 and offer 2 values, aligned to the offer 2 onset. Shading indicates the upper and lower bounds of the 99% credible interval. The first vertical line indicates the appearance of the second offer. The second vertical line indicates the end of the offer 2 epoch, after which the second offer disappears. This plot also confirms our findings in the main text. It also shows that the mutual inhibition signal fades and offers are no longer encoded in opposing formats as the trial transitions into the choice epoch. (TIF) [file pbio.2003091.s014.tif]
